# Role of dopamine and gray matter density in aging effects and individual differences of functional connectomes

**DOI:** 10.1007/s00429-020-02205-4

**Published:** 2021-01-09

**Authors:** Benjamín Garzón, Martin Lövdén, Lieke de Boer, Jan Axelsson, Katrine Riklund, Lars Bäckman, Lars Nyberg, Marc Guitart-Masip

**Affiliations:** 1 Aging Research Center, Karolinska Institutet, Stockholm, Sweden; 2 Department of Radiation Sciences, Umeå University, Umeå, Sweden; 3 Umeå Center for Functional Brain Imaging, Umeå University, Umeå, Sweden; 4 Department of Integrative Medical Biology, Umeå University, Umeå, Sweden; 5 Max Planck UCL Centre for Computational Psychiatry and Ageing Research, University College London, London, UK; 6 Aging Research Center, Tomtebodavägen 18A, 171 65 Solna, Sweden

**Keywords:** Dopamine, Aging, Functional connectome, fMRI, PET

## Abstract

With increasing age, functional connectomes become dissimilar across normal individuals, reflecting heterogenous aging effects on functional connectivity (FC). We investigated the distribution of these effects across the connectome and their relationship with age-related differences in dopamine (DA) D1 receptor availability and gray matter density (GMD). With this aim, we determined aging effects on mean and interindividual variance of FC using fMRI in 30 younger and 30 older healthy subjects and mapped the contribution of each connection to the patterns of age-related similarity loss. Aging effects on mean FC accounted mainly for the dissimilarity between connectomes of younger and older adults, and were related, across brain regions, to aging effects on DA D1 receptor availability. Aging effects on the variance of FC indicated a global increase in variance with advancing age, explained connectome dissimilarity among older subjects and were related to aging effects on variance of GMD. The relationship between aging and the similarity of connectomes can thus be partly explained by age differences in DA modulation and gray matter structure.

**Supplementary Information:**

The online version contains supplementary material available at 10.1007/s00429-020-02205-4.

## Introduction

Aging produces changes in the coupling between spontaneous oscillations of the blood oxygen level-dependent (BOLD) signal (Andrews-Hanna et al. 2007; Damoiseaux et al. 2008; Ferreira et al. 2016), which is commonly termed functional connectivity (FC). FC decreases in advanced age within the default-mode network (Mevel et al. 2013) as well as within the cingulo-opercular and fronto-parietal circuitries, while in visual and somatomotor networks both stability (Chan et al. 2014; Geerligs et al. 2015a) and reductions (Mowinckel et al. 2012; Stumme et al. 2020) have been found. FC increases have also been reported between left and right hippocampus (Salami et al. 2014, 2016). These results indicate that the effects of aging are heterogeneous across connections, and suggest the existence of a shift in the pattern of functional connections with increasing age, rather than a global, homogeneous change.

The set of functional connections for all possible pairs of regions from a parcellation covering the brain comprehensively constitutes a functional connectome. This can be represented as a graph consisting of a set of nodes (regions) linked by edges (functional connections), and its FC values can be arranged in a (symmetric) matrix. Given two connectomes, we can define Pearson’s correlation coefficient between the subjects’ sets of FC values as a measure of their similarity. A substantial portion of the connectome is stable and unique to each individual (Finn et al. 2015; Geerligs et al. 2015b): using this measure of similarity, two measurements (performed on different days) of the functional connectome of a certain individual tend to be more similar than the connectomes of different individuals, allowing to identify individuals based solely on their connectome with considerable accuracy (Finn et al. 2015).

Aging is accompanied by a loss of similarity between the connectomes of younger and older adults as well as between the connectomes of older subjects (Geerligs et al. 2015b). In view of the aforementioned heterogeneity of FC across connections, assessing and mapping the magnitude of age-related differences in mean FC and interindividual FC variance across individuals can help quantify the contribution of each connection to these similarity reductions. This will facilitate the understanding of the source of age-related connectome dissimilarity and the assessment of its implications for cognitive function.

**Fig. 1:**
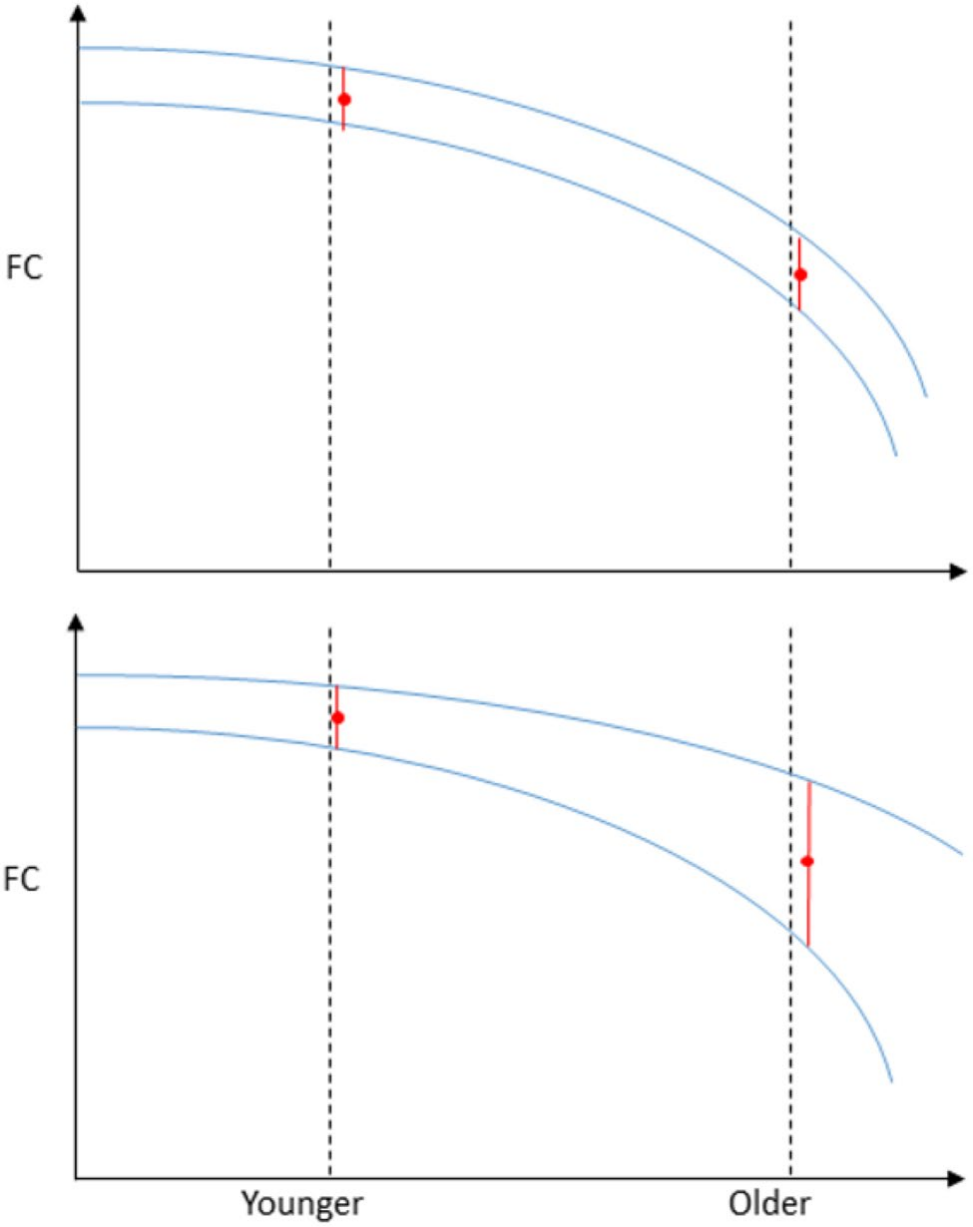
Hypothetical trajectories of FC in two different connections. We quantified between-group differences in both mean FC (indicated by the red dots) and standard deviation (indicated by the red segments arising from the red dots) as two dimensions to characterize aging effects. When sampled cross-sectionally, parallel trajectories (top) will result in similar variance between younger and older participants, whereas a spread in trajectories will result in increased variance with age (bottom)

In the hypothetical case that we were able to sample the individual lifespan trajectories of a neurobiological measure at two different ages (e.g., younger and older adulthood), a group difference in the mean of the variable of interest might be observed, with its magnitude depending on the aggregate evolution of the individual time courses. In addition, unless these trajectories evolved in a parallel fashion, a difference in the variance of the two groups would be likely to be observed, with the magnitude being conditional on the spread of the trajectories (Fig. 1). An increase with age in between-subject variance can be noteworthy because, assuming that this increase is not mainly driven by cohort differences or selection bias, it may reflect maintenance (Nyberg et al. 2012; Fandakova et al. 2015) in some subjects and not in others due to the existence of protective and/or risk factors related to the neurobiological measure. In the present work, we studied how aging effects (i.e., age differences, under the assumption that they are primarily reflecting aging processes) on mean and variance of FC are distributed across the connectome and relate to the age dependence observed in connectome similarity.

Which are the neurobiological mechanisms behind the increasing connectome dissimilarity with age? Among the host of changes occurring in the human brain as part of normal aging, the existence of dopaminergic deficits is one of the main causal hypotheses for age-related differences in FC (Ferreira et al. 2016). Pharmacological and neuroimaging research has associated dopamine (DA) and functional connectivity in specific circuits: the cortico-striato-thalamic system (Honey et al. 2003), caudate-medial temporal lobe (Nyberg et al. 2016), fronto-parietal pathways (Rieckmann et al. 2011), and fronto-striatal pathways (Wallace et al. 2011). In prefrontal-cortex in particular, the role of DA may be to modulate the signal-to-noise ratio of neuronal firing (Servan-Schreiber et al. 1990), facilitating the stabilization of neural representations (Durstewitz and Seamans 2008). On the other hand, there is a well-established, mainly from cross-sectional studies, gradual loss of DA markers (DA transporter, and D1 and D2 receptors) from early through late adulthood (Bäckman et al. 2010). Further, computational modelling has linked age-related DA deficits with increases in individual differences in activation patterns and functional neural architecture (Li and Lindenberger 1999; Li et al. 2001; Li and Sikström 2002), and pharmacological manipulations of dopaminergic neurotransmission modulate the organization of large-scale functional networks (Cole et al. 2013; Carbonell et al. 2014). Therefore, DA decline is an obvious candidate driving some of the age-related decreases in connectome similarity highlighted above. Another candidate to explain these differences is the conspicuous reduction in volume of cortical and subcortical gray matter structures known to occur in healthy aging, which shows marked regional heterogeneity (Raz et al. 2005; Kennedy et al. 2009; Groves et al. 2012).

In a sample of younger (19–32 years) and older (66–72 years) healthy subjects, we mapped aging effects on the mean and variance of FC of over 30,000 functional connections. Using this decomposition, we sought to identify the individual contribution of the different anatomical connections and functional networks to the patterns of connectome similarity loss with increasing age. Positron emission tomography (PET) imaging with the D1 receptor ligand [11C] SCH23390, a tracer allowing quantification of D1 receptor availability in cortical and subcortical regions, was used to probe the association between age-related differences in regional D1 receptor availability and age-related differences in FC across connections. Likewise, we assessed the association between age-related differences in gray matter density (GMD) measured with structural MRI and the corresponding regional age-related differences in FC. The FC analyses were done separately using datasets from three fMRI experiments performed on the same participants (two cognitive tasks and a resting-state acquisition).

## Materials and methods

### Study participants

Participants were 30 younger (mean age = 24.2, SD = 3.4 years, 13 men and 17 women) and 30 older (mean age = 70.8, SD = 2.7 years, 18 men and 12 women) healthy volunteers who were recruited through local media advertisements. This sample was initially acquired to investigate age-related differences in a number of facets of decision-making. The main findings of the analyses of the behavioral data and fMRI tasks have been reported elsewhere (de Boer et al. 2017, 2019), while in the present study we focus on connectivity, neuromodulatory and structural measures. Sample size and power were calculated considering behavioral and task-based fMRI effects in similar studies of decision-making (Guitart-Masip et al. 2012, 2014; Chowdhury et al. 2013).

Participants were paid a fixed amount for their participation in the study, and additionally a variable amount that depended on their performance in the battery. Based on self-reports, all participants were free of neuropsychiatric disorders, other significant medical conditions and current or previous substance abuse. The Regional Research Ethics Committee in Umeå (Sweden) approved the research protocol of the study. All participants provided written informed consent and underwent structural and functional MRI and PET scanning.

### MRI data acquisition

Subjects were imaged on a Discovery MR750 3 T scanner (General Electric, Milwaukee, WI, USA), equipped with a 32-channel phased-array head coil. T_1_-weighted 3D FSPGR images were obtained with a 1 × 1 × 1 mm^3^ voxel size (TI = 450 ms, TR = 8.17 ms, TE = 3.19 ms, FoV = 25 cm, flip angle of 12°). Functional scans were acquired with a gradient echo-planar imaging sequence (TR = 2000 ms, TE = 30 ms, FoV = 25 cm, 37 axial slices, flip angle of 80°, slice thickness = 3.4 mm with 0.5 mm spacing) with voxel size of 2 × 2 × 3.9 mm^3^. The first ten EPI volumes were discarded to ensure that steady-state tissue longitudinal magnetization was reached. Three functional acquisitions were performed in the same scanning session, one in resting state (RS) and two during decision-making tasks in which subjects responded to visual stimuli by pressing a button to obtain rewards. For the RS scan, subjects were instructed to remain awake with their eyes open and fixated on a cross (170 volumes, 5.7 min). In the second acquisition subjects completed the two–armed bandit (TAB) task (660 volumes, 22 min; (Chowdhury et al. 2013). In the third acquisition, subjects performed the go-nogo (GNG) valenced task (630 volumes, 21 min; (Guitart-Masip et al. 2011). There were differences in performance between younger and older adults in the two tasks. The aging effects in the TAB task have already been reported (de Boer et al. 2017) and the effects in the GNG are in preparation.

### MRI data preprocessing

The data were preprocessed with SPM12 (http://www.fil.ion.ucl.ac.uk/spm) and the Data Processing Assistant for Resting-State fMRI: Advanced Edition (DPARSFA, version 2.3) (Yan 2010), running on MATLAB R2017b (Mathworks, Natick, USA).

Structural scans were segmented into gray matter, white matter, and cerebrospinal fluid (CSF) (Ashburner and Friston 2005). The Diffeomorphic Anatomical Registration using Exponentiated Lie Algebra (DARTEL) algorithm (Ashburner 2007) was used to create a study-specific anatomical template, followed by its affine transformation to the Montreal Neurological Institute (MNI) template. Segmentation of the cerebellum was obtained using Freesurfer 5.3 (Fischl et al. 2002), to be used in the subsequent analysis of PET data.

Functional scans were first slice-time corrected and realigned to correct for inter-scan head motion. To obtain a single measure of overall motion, at each time-point we calculated the framewise displacement (FD) from these parameters, as described in (Power et al. 2012). Average signal time-series were extracted from masks of white matter and CSF obtained in the aforementioned segmentation procedure. To remove non-neural confounds, the 6 motion parameters (translations and rotations) and their temporal derivatives, the square of these 12 time-courses and the white matter and CSF time-series, were regressed out from the signal of the corrected functional images at each voxel (Satterthwaite et al. 2013). We did not regress out the task-evoked activity in accordance with several recent studies (Finn et al. 2015, 2017; Rosenberg et al. 2015; Lebedev et al. 2018) and to underscore that even when considering the correlations produced by it, there are still noticeable similarities between experiments.

There is no clear consensus regarding whether global signal regression (GSR) should be used as a strategy to reduce the effect of motion on connectivity measures (Murphy and Fox 2017). While this step may minimize the relationship between motion and connectivity, it may also introduce artifacts and remove common fluctuations of neural origin (Ciric et al. 2017; Murphy and Fox 2017). Therefore, we produced two preprocessed datasets, one with and another one without GSR, to ensure that our results did not depend on either motion or the use of GSR. The images were band-pass filtered in the band 0.009–0.1 Hz to reduce low-frequency drifts and high-frequency physiological as well as non-biological signals (Biswal et al. 1995). The processed functional scans were coregistered to the structural ones, normalized to MNI space using the transformations estimated previously and resampled to 2 × 2 × 2 mm^3^. Next, the images were smoothed with a Gaussian kernel of 4 mm full width at half maximum (FWHM). Finally, the data were scrubbed by removing frames with FD above a certain threshold for their corresponding time point, as well as the previous and the two next images (Power et al. 2012). For the GNG and TAB we used an FD threshold of 0.3 mm and only participants with 300 or more images left after scrubbing were considered for further analyses. For the RS data, which had considerably shorter duration, we used a slightly more lenient FD threshold of 0.4 mm and kept subjects with more than 130 images left after scrubbing, as the criteria used for the GNG and TAB datasets would otherwise have left too little data for analysis.

Mean FD after scrubbing was largely reduced in all cases, as was the difference between mean FD for the two age groups. It was still significantly different between younger and older adults for the GNG task (two sample *t* test, *t* = − 2.17, *p* = 0.04), but not for the TAB (two sample *t* test, *t* = − 1.30, *p* = 0.20) or the RS dataset (two sample *t* test, *t* = 1.54, *p* = 0.13). Whole-sample mean FD prior to scrubbing was significantly or nearly significantly larger for the tasks than for the RS data (two sample *t* test, GNG-RS: *t* = 2.07, *p* = 0.04, TAB-RS: *t* = 1.90, *p* = 0.06), but not significantly different between the two tasks (GNG-TAB: *t* = 0.01, *p* = 0.99).

### Computation of functional connectomes

Average preprocessed signal time-series were extracted for each of 278 regions of interest (ROIs) from a functionally defined parcellation (Shen et al. 2013, Supplementary Fig. 1A). We excluded ROIs with large signal loss due to susceptibility artifacts (Geerligs et al. 2015b). For each subject, the average of all functional images was obtained and a mask was created by thresholding this average at 20% of mean signal intensity. Seventeen ROIs overlapping this mask in less than 50% of the ROI volume were excluded, leaving 261 ROIs with sufficient signal in the three fMRI acquisitions. The excluded ROIs were located in temporal, orbitofrontal and occipital regions and are shown in Supplementary Fig. 1B. A functional connectome was computed as the set of Pearson’s correlation coefficients $r$ between time-series of all pairs of nodes after applying the Fisher transform $z = \mathrm{arctanh}(r)$ to render these into z-scores (Fisher 1921), since subsequent analyses assumed a normal distribution for FC values. A connectome was then represented as a symmetrical matrix of 261 × 261 entries, totaling 33,930 different connections (node pairs). We computed a functional connectome for each subject and each dataset (GNG/TAB/RS) separately, and the subsequent analyses were performed independently for each dataset.

### Similarity between functional connectomes

We computed the similarity between connectomes as Pearson’s correlation coefficient (as FC values were normally distributed for the reason explained above) between the entries in the upper triangular part of the corresponding matrices, excluding the diagonal. One value of similarity was computed for each pair of subjects in the sample and displayed in a matrix of subjects x subjects, ordered by age. To display the degree of similarity between the observations in the dataset, we used multidimensional scaling (MDS, estimated with the R function cmdscale), a technique that allows visualization in a two-dimensional plot of multidimensional observations by preserving the dissimilarity (distance) in their original space.

### Voxel-based morphometry

The gray matter segments computed for preprocessing the functional data were multiplied by the Jacobian of the non-linear transformations estimated by the DARTEL procedure (also known as modulation) to produce voxel-based morphometry maps, which approximate local GMD (Ashburner and Friston 2000). Average GMD was then calculated for each node of the parcellation.

### PET data acquisition

PET images were obtained using a Discovery 690 PET/CT (General Electric, WI, US) scanner. A low-dose helical CT scan (20 mA, 120 kV, 0.8 s/revolution) provided data for PET attenuation correction. Participants were injected with a bolus of 200 MBq [11C]SCH 23,390, a radioligand that binds to dopamine D1 receptors. A 55 min dynamic acquisition commenced at time of injection (9 frames × 2 min, 3 frames × 3 min, 3 frames × 4.20 min, 3 frames × 5 min). Attenuation-, scatter- and decay-corrected 256 × 256-pixel transaxial PET images were reconstructed to a 25 cm field-of-view employing the Sharp IR algorithm (6 iterations, 24 subsets, 3.0 mm Gaussian post filter). Sharp IR is an advanced version of the OSEM method for improving spatial resolution, in which detector system responses are included (Ross and Stearns 2010). The FWHM resolution is 3 mm. The protocol resulted in 47 tomographic slices per time frame, yielding 0.977 × 0.977 × 3.27 mm^3^ voxels. Images were decay-corrected to the start of the scan and de-identified using dicom2usb (http://dicom-port.com/). To minimize head movement during the imaging session, the subject’s head was fixated with an individually fitted thermoplastic mask (Positocasts Thermoplastic; CIVCO medical solutions, IA, US).

### PET data preprocessing

PET data were preprocessed with in-house-developed software (imlook4d version 5.00, https://dicom-port.com/product/imlook4d/). The PET time series were coregistered to the structural scans with the FMRIB's Linear Image Registration Tool (FLIRT, (Jenkinson et al. 2002)) from the FMRIB Software Library 5.0.9 (FSL, http://fsl.fmrib.ox.ac.uk/). The warps obtained from the DARTEL normalization procedure were then used to coregister the 278 ROIs from the parcellation previously described to the PET time series that were aligned with the structural scans. Binding potential (BP) was calculated by subtracting 1 to distribution volume ratios (DVR) obtained by applying the Logan method (Logan et al. 1990) with time window between 18 and 55 min to average time-activity curves extracted from all voxels in each ROI. A cerebellum ROI (previously segmented from the structural scans) was used as reference, because its tissue is devoid of DA D1 receptors (Hall et al. 1994). Given that the cerebellum was used as a reference for the calculation of BP, ROIs in this region were excluded from later analyses involving this measure.

### Modelling of age-related effects on mean and variance of imaging measures

We modelled aging effects on mean and interindividual variance of imaging data ${y}_{s}^{j}$ from subject *s*, where ${y}_{s}^{j}$ can be the FC estimate in connection $j$, BP estimate in region $j$ or GMD estimate in region $j$ ($j$ indexes regions or connections, depending on the variable being modelled, and represents a superscript, not an exponent). The data were modelled as normally distributed with mean and the log of the standard deviation as linear functions of ${a}_{s}$ (the participant’s age referenced to 20 years), plus a term ${\mathrm{FD}}_{s}$ accounting for the effect of head motion on the imaging measures.

1 $${y}_{s}^{j}= {z}_{s}^{j}+ \rho {\cdot \mathrm{FD}}_{s},$$

2 $${z}_{s}^{j} \sim N\left({\mu }_{s}^{j}\left({a}_{s}\right), {\sigma }_{s}^{j}\left({a}_{s}\right)\right),$$

3 $${\mu }_{s}^{j}\left({a}_{s}\right) = {\alpha }_{\mu }^{sj}+ {\beta }_{\mu }^{sj}\cdot {a}_{s},$$

4 $$\mathrm{log}{\ \sigma }_{s}^{j}\left({a}_{s}\right) = {\alpha }_{\sigma }^{sj}+ {\beta }_{\sigma }^{sj}\cdot {a}_{s}.$$

The value of ${a}_{s}$ is referenced so that the values of the intercepts $\alpha$ can be easily interpreted as corresponding to 20-years old subjects, and the values of the slopes $\beta$ the annual increment or decrement in mean or log standard deviation above the intercept value. The log transformation in Eq. 4 ensures positivity, i.e., ${\sigma }_{s}^{j}\left({a}_{s}\right) = {\mathrm{exp}(\alpha }_{\sigma }^{sj}+ {\beta }_{\sigma }^{sj}\cdot {a}_{s})$. Positive (negative) values of ${\beta }_{\sigma }$ imply that the standard deviation at 20 years of age, ${\mathrm{exp}(\alpha }_{\sigma }^{sj}),$ increases (decreases) by a factor of $\mathrm{exp}({\beta }_{\sigma }^{sj})$ for each additional year. Head motion during functional scans has been shown to affect not only FC measures (Power et al. 2012) but also to behave like an individual trait (Van Dijk et al. 2012) that approximates movement during structural scans, known to affect structural estimates (Reuter et al. 2015; Savalia et al. 2017). In summary, we propose a model where both mean and variance of FC are dependent on age (controlling for in-scanner head motion), with the aim of estimating how mean and variance vary with age across different brain connections. As Fig. 1 illustrates, these parameters should be informative about the trends and spread of the underlying trajectories of FC, always keeping in mind that they are obtained from cross-sectional data.

The same model structure was used to model FC, BP, and GMD. We inferred parameter values using maximum a posteriori estimation with weakly informative priors that were normal and symmetric around 0 to avoid biases toward a particular direction (e.g., when estimating the effects of aging $\beta$). Model fitting was done with the *optimizing* function from the rstan package 2.17.3 (http://mc-stan.org/).

Finally, with the purpose of assessing the consistency of the estimates across fMRI datasets, for each possible pair of experiments we computed Pearson’s correlation coefficients between the values of ${\beta }_{\mu }$ (or ${\beta }_{\sigma }$).

### Anatomical distribution of connection parameters

To facilitate the visualization and comprehension of ${\beta }_{\mu }$ and ${\beta }_{\sigma }$*,* we partitioned the parcellation in the brain stem and nine structures in each hemisphere (thalamus, putamen, caudate, insula, cerebellum, and the temporal, parietal, frontal and occipital lobes). The Atlasquery tool from FSL was used to calculate the probability of a certain ROI being a member of the different labelled regions in the MNI Structural Atlas, or the Harvard–Oxford Subcortical Structural Atlas for the brain stem label. Each ROI was assigned to the region with the highest probability. Then we plotted the values of ${\beta }_{\mu }$ and ${\beta }_{\sigma }$ laid out in respective matrices arranged by these anatomical regions.

We also partitioned the parcellation according to a decomposition in 20 networks (Laird et al. 2011) retrieved from http://www.brainmap.org. BrainMap (Fox et al. 2005) is an online database of brain activation coordinates and structured metadata describing the experimental conditions from a large number of neuroimaging studies. The database has been used to yield an extensive set of activation maps, decomposed via independent component analysis into a set of task-based co-activation networks which can be related to particular cognitive domains and mimic the intrinsic connectivity networks (ICNs) that can be measured in resting-state acquisitions (Smith et al. 2009). The original dataset of 20 co-activation network maps contains two artifactual maps, which were removed. Detailed descriptions and illustrations of the 18 co-activation network maps can be found in (Smith et al. 2009) and (Laird et al. 2011); in the present article we follow the same numbering as in those publications (see Supplementary Table 1 for a short description). For each ROI in the parcellation, we extracted the average value within the ROI of each of the co-activation network maps and multiplied it by ROI size, producing a score for each network-ROI combination. We then assigned each ROI to the network with highest score. We plotted the values of aging effects ${\beta }_{\mu }$ and ${\beta }_{\sigma }$ laid out in respective matrices arranged by these 18 networks.

### Associations between FC, BP, and GMD

We sought to link aging effects in FC, BP and GMD. First, for each node in the parcellation included in the analysis of aging effects on FC, we computed the average value of ${\beta }_{\mu }$ and ${\beta }_{\sigma }$ for all connections containing that node (ROI), henceforth referred to respectively as ‘average nodal ${\beta }_{\mu }$’ and ‘average nodal ${\beta }_{\sigma }$’, thereby obtaining an average aging effect for the mean and the standard deviation of FC for each node. As explained previously, we also estimated values of ${\beta }_{\mu }$ and ${\beta }_{\sigma }$ for both BP and GMD in each node of the same parcellation. Then we computed Pearson’s correlation coefficients between average ${\beta }_{\mu }$ for FC and ${\beta }_{\mu }$ for either BP or GMD and plotted these relationships, and we did likewise for ${\beta }_{\sigma }$. We also set up linear models with average nodal ${\beta }_{\mu }$ for FC as dependent variable and both ${\beta }_{\mu }$ for BP and ${\beta }_{\mu }$ for GMD as independent variables to check whether these regressors accounted for independent portions of the variance in the dependent variable.

All statistical analyses were conducted with R version 3.5.1 (https://www.r-project.org/).

## Results

### MRI and PET data preprocessing

The three datasets (GNG/TAB/RS) were preprocessed with the same standard pipeline to obtain three FC matrices (representing functional connectomes) for each subject. Some subjects failed to meet the criteria for acceptable levels of motion in the fMRI experiments studying FC and were excluded from the analyses (10 for the TAB, 3 for the GNG and 5 for the RS experiment). The characteristics of the included subjects are presented in Table 1. Henceforth, for the sake of brevity we mainly present results for the GNG task dataset unless indicated otherwise (for this task had the smallest number of excluded subjects), but we also comment on the consistency across experiments for the main findings, principally when there was disagreement between them. The results presented correspond to the datasets preprocessed without GSR, and in the last section we make remarks about discrepancies arising when using GSR.

**Table 1:** Demographic characteristics for the whole sample, the fMRI acquisitions, and the PET assessment

		All	TAB	GNG	RS	D1 PET
Number of subjects		60	50	57	55	58
Younger	Total (M/F)	30 (13/17)	28 (12/16)	30 (13/17)	30 (13/17)	30 (13/17)
	Age mean (SD)	24.2 (3.4)	24.4 (3.5)	24.2 (3.4)	24.2 (3.4)	24.2 (3.4)
Older	Total (M/F)	30 (18/12)	22 (14/8)	27 (17/10)	25 (17/8)	28 (17/11)
	Age mean (SD)	70.8 (2.7)	70.9 (2.6)	70.8 (2.7)	70.9 (2.7)	71.0 (2.7)

*TAB* two-arm bandit task, *GNG* go-nogo task, *RS* resting-state

BP values quantifying D1 receptor availability were obtained for all except two participants that did not complete the PET scans. The structural scans were processed using a standard voxel-based morphometry pipeline, yielding voxelwise maps of GMD of adequate quality for all subjects.

### Similarity between functional connectomes

**Fig. 2:**
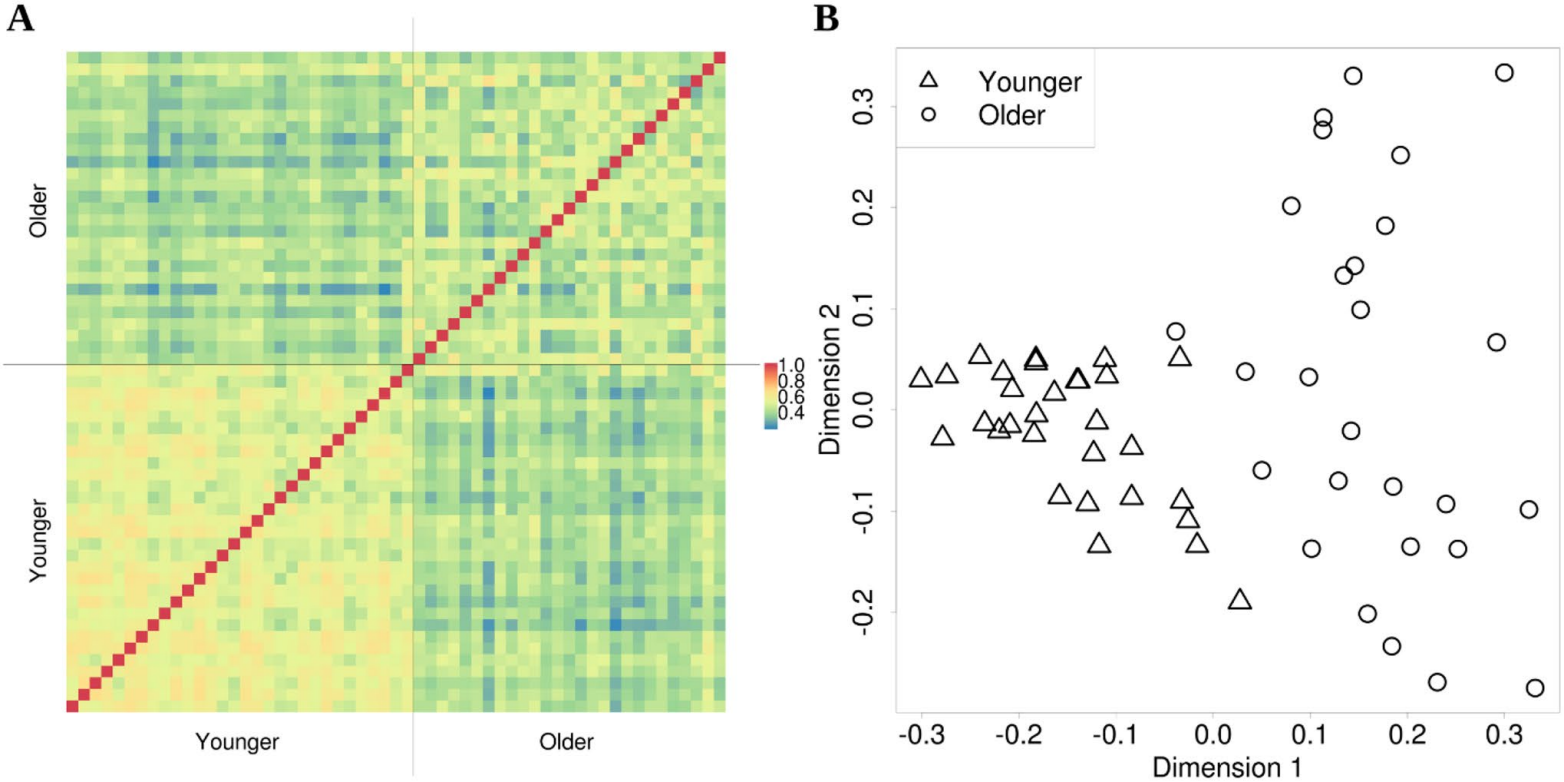
Similarity between connectomes in younger and older subjects. **a** Similarity matrix for pairs of subjects in the sample, showing an age-dependent pattern of similarity. Each entry corresponds to the connectome similarity between two subjects, defined as Pearson’s correlation coefficient between the FC values of all connections in the connectomes of those two subjects. **b** Multidimensional scaling visualization of those subjects according to their similarity pattern. In this representation, the magnitude in the axes is arbitrary, but the distance between the points in the scatterplot indicates the degree of dissimilarity between observations (without the values in each of the two axes necessarily having a specific interpretation). The younger and older groups are clearly separated, and at the same time older subjects are farther apart between them than younger subjects between them, implying that the connectomes of the older participants are more dissimilar

The matrix in Fig. 2a shows the similarity (Pearson’s correlation coefficients) between the functional connectomes of all pairs of subjects for the GNG task, and the scatterplot in Fig. 2b portrays the MDS representation of the dataset. MDS is a technique that allows visualization of multidimensional observations in a two-dimensional plot by preserving their dissimilarity (distance) in the original space (Hout et al. 2016). Similarities for pairs of older subjects tended to be lower than for pairs of younger subjects, resulting in larger spread for the older with respect to the younger subjects in the MDS representation. Similarities between younger and older subjects also tended to be lower than for pairs of younger or older subjects, clearly separating the two age groups in the MDS visualization. The difference between the average younger-younger similarity and the average older-older similarity was 0.08 (two sample *t* test, *t* = 17.0, *p* < 2*e *− 10). The difference between average older-older similarity and older-younger similarity was 0.05 (two sample *t* test, *t* = 9.93, *p* < 2*e *− 10).

### Age-related effects on mean and variance of FC

We estimated aging effects on mean (${\beta }_{\mu }$) and standard deviation (${\beta }_{\sigma }$) of FC in each connection. ${\beta }_{\mu }$ values tended to be negative (GNG: 64.0% of connections; TAB: 67.2%; RS: 62.9%), indicating that in most connections mean FC was lower in older than in younger subjects, whereas ${\beta }_{\sigma }$ values were overall positive (GNG: 76.2% of connections; TAB: 75.5%; RS: 74.6%), suggesting larger between-person differences in FC among older adults.

The correlation between the estimates of the GNG and TAB tasks was high for ${\beta }_{\mu }$ and moderate for ${\beta }_{\sigma }$, which indicates that the aging effects we observed are relatively independent of the task the subjects performed during scanning (Table 2). The correlation between the estimates of the GNG and RS experiments was lower (presumably due to the considerably shorter length of the RS dataset), and comparable to the correlation between TAB and RS estimates.

**Table 2:** Consistency of estimates across experiments

	GNG-TAB	TAB-RS	GNG-RS
Without GSR			
${\beta }_{\mu }$	0.89	0.69	0.69
${\beta }_{\sigma }$	0.55	0.28	0.29
With GSR			
${\beta }_{\mu }$	0.90	0.68	0.70
${\beta }_{\sigma }$	0.53	0.29	0.32

The entries in the table correspond to Pearson coefficients for the correlation between the mean posterior estimates of ${\beta }_{\mu }$ and ${\beta }_{\sigma }$(aging effects for FC for individual connections) in each pair of experiments. For clarity p values are not shown, as all tests were significant (*p* < 1*e *− 3). *TAB* two-arm bandit task, *GNG* go-nogo task, *RS* resting-state

### Anatomical distribution of connection parameters

**Fig. 3:**
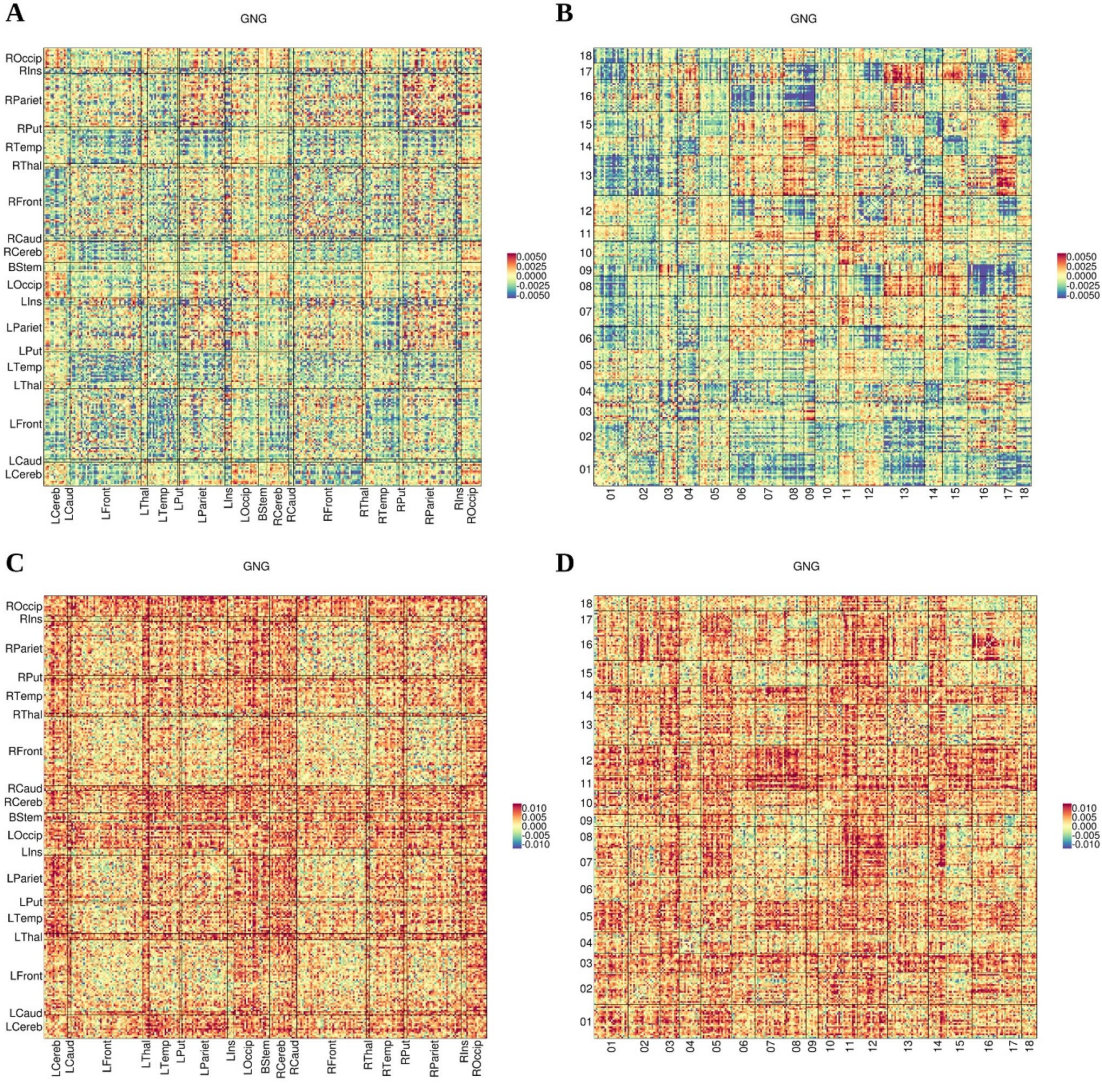
Estimates of aging effects across the connectome for data processed not using GSR. For each connection between a pair of nodes in the parcellation, we show the estimates of ${\beta }_{\mu }$ (aging effects on mean FC; **a**, **b**) and ${\beta }_{\sigma }$ (aging effects on standard deviation of FC; **c**, **d**) for the GNG task without GSR. In **a** and **c**, connections are laid out in matrices that group the corresponding nodes in 19 anatomical regions: brain stem (BStem), bilateral thalamus (LThal/RThal), putamen (LPut/RPut), caudate (LCaud/RCaud), insula (LIns/RIns), cerebellum (LCereb/RCereb), and the temporal (LTemp/RTemp), parietal (LPariet/RPariet), frontal (LFront/RFront) and occipital (LOccip/ROccip) lobes. In **b** and **d**, the matrices are arranged by the networks in the 20-network BrainMap decomposition (see Supplementary Table 1 for numbering and a short description of the networks). The estimates for the other two experiments are displayed in Supplementary Figure S3 (TAB) and Supplementary Figure S4 (RS)

Figure 3a, c summarizes the anatomical distribution of aging effects on FC, ${\beta }_{\mu }$ and ${\beta }_{\sigma }$, with connections grouped according to their endpoint anatomical region, for the GNG experiment (see Supplementary Figs. 3 and 4 for the TAB and RS experiments, respectively). Fronto-temporal, fronto-cerebellar and temporo-parietal connections had predominantly negative ${\beta }_{\mu }$, whereas a considerable amount of intra-parietal connections showed large positive differences between age groups. The inspection of the values of ${\beta }_{\sigma }$ revealed a general increase in FC variability with age with occipital, thalamic and cerebellar connections presenting larger age-related effects on variability compared with connections arising from other regions. There were no obvious hemispheric asymmetries.

An alternative arrangement according to the 18 BrainMap networks (Fig. 3b, d) showed that connections between networks 09 (superior parietal) and 16 (transverse temporal), or between networks 01 (limbic and medial temporal) and 13 (default mode) presented with mostly negative ${\beta }_{\mu }$ values, whereas connections between networks 13 (default mode) and 17 (pre/postcentral) were mostly positive. The largest age differences in mean FC were found in inter-network rather than intra-network connections. The largest values of ${\beta }_{\sigma }$ were concentrated in connections from networks 05 (cerebellum-brainstem) and 12 (medial visual), and especially between 08 (sensorimotor) and 11 (lateral visual) or 12 (medial visual).

### Associations among aging effects in FC, D1 BP, and GMD

From the PET and structural scans, we calculated average BP and GMD values in each ROI of the parcellation, and modelled aging effects on mean and standard deviation of BP and GMD as we had done for the FC values. ${\beta }_{\mu }$ was negative for both GMD and BP in all ROIs. ${\beta }_{\sigma }$ was positive in 56.8% of ROIs for GMD and 79.9% of them for BP.

**Fig. 4:**
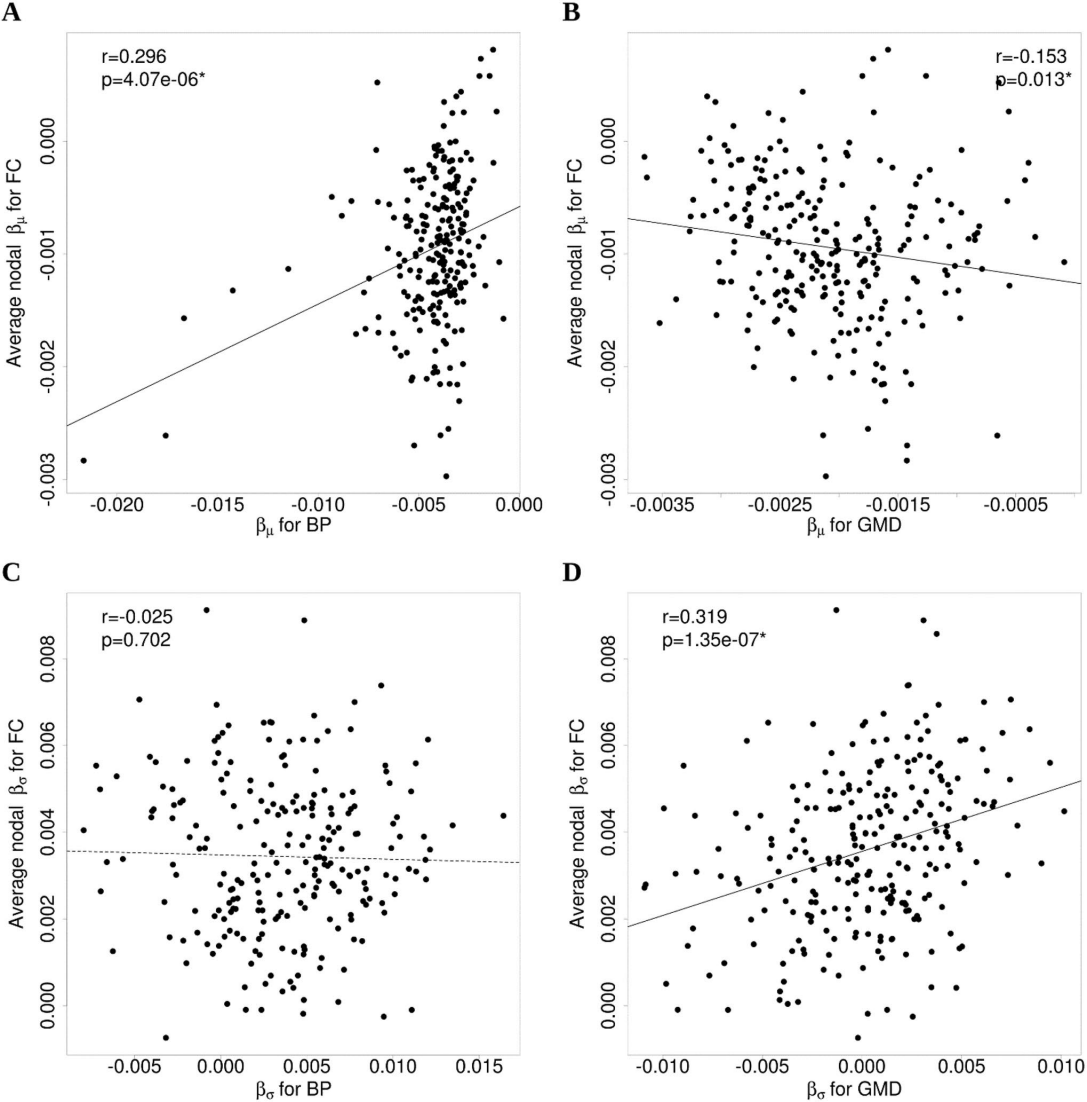
Association between FC, BP and GMD aging effects. **a** Association, across 244 parcellation regions (regions within the cerebellum were excluded from this analysis), between average nodal aging effects on mean FC and aging effects on mean DA D1 BP. **b** There was a trend for a negative association between average nodal aging effects on mean FC and aging effects on GMD across the 261 parcellation regions. **c** There was no correlation between average nodal aging effects on standard deviation of FC and aging effects on standard deviation of BP. **d** Association between average nodal aging effects on standard deviation of FC and aging effects on standard deviation of GMD. All these plots correspond to the GNG dataset, but similar results were found for the other experiments. *BP* binding potential, *GMD* gray matter density, *FC* functional connectivity. *Significant association (*p* < 0.05)

We investigated whether the aging effects in FC were related to those in BP and GMD across ROIs. Average nodal ${\beta }_{\mu }$ for FC was significantly, positively correlated with ${\beta }_{\mu }$ for BP (GNG: *r* = 0.30, *p* = 4*e *− 6; TAB: *r* = 0.20, *p* = 1*e *− 3; RS: *r* = 0.28, *p* = 1*e *− 5), indicating that larger (negative) age differences in FC were associated with larger (negative) age differences in D1 BP. The ROIs with the largest age-related BP losses (${\beta }_{\mu }$ < − 0.01) were bilateral putamen and caudate, as well as left thalamus. However, the association was not only driven by these regions, as it was still statistically significant after removing their values from the analysis (GNG: *r* = 0.18, *p* = 5*e* − 3; TAB: *r* = 0.13, *p* = 0.05; RS: *r* = 0.14, *p* = 0.03). Average nodal ${\beta }_{\mu }$ for FC was negatively correlated with ${\beta }_{\mu }$ for GMD for the GNG (*r* =  − 0.15, *p* = 0.01; Fig. 4b) and RS (*r* =  − 0.13, *p* = 0.04) datasets, with a trend toward significance for the TAB dataset (*r* =  − 0.11, *p* = 0.09). When combining ${\beta }_{\mu }$ for D1 BP and ${\beta }_{\mu }$ for GMD in one regression model to predict average nodal ${\beta }_{\mu }$ for FC, the regression coefficients for ${\beta }_{\mu }$ for BP were still statistically significant (GNG: standardized coefficient = ${\beta }_{\mu }$18*e *− 5, *p* = 2*e *− 5; TAB: standardized coefficient = ${\beta }_{\mu }$15*e *− 5, *p* = *4e *− 3; RS: standardized coefficient = ${\beta }_{\mu }$14*e *− 5, *p* = 4*e *− 5), and therefore the association between aging effects (on FC and BP) cannot be explained by aging effects on GMD. The coefficients of these models are presented in Table 3.

**Table 3:** Regression models relating aging effects in mean FC with aging effects in mean BP and mean GMD across regions

	GNG	TAB	RS
Without GSR			
Average nodal ${\beta }_{\mu }$ for BP	***18e − 5 (p = 2e − 5)***	***15e − 5 (p = 4e − 3)***	***14e − 5 (p = 4e − 5)***
Average nodal ${\beta }_{\mu }$ for GMD	− 8*e* − 5 (*p* = 0.0517)	− 6*e* − 5 (*p* = 0.26)	− 7*e* − 5 (*p* = 0.051)
With GSR			
Average nodal ${\beta }_{\mu }$ for BP	***8e − 5 (p = 0.008)***	7*e* − 5 (*p* = 0.0552)	***6e − 5 (p = 0.001)***
Average nodal ${\beta }_{\mu }$ for GMD	*** − 27e − 5 (p < 1e − 10)***	*** − 30e − 5 (p < 1e − 10)***	*** − 21e − 5 (p < 1e − 10)***

The dependent variable was average nodal ${\beta }_{\mu }$ for FC, and the independent variables were ${\beta }_{\mu }$ for BP and ${\beta }_{\mu }$ for GMD. The table shows the corresponding standardized regression coefficients and p values. Coefficients significantly different from zero (*p* < 0.05) are marked in bold font

*TAB* two-arm bandit task, *GNG* go-nogo to task, *RS* resting-state

The association between average nodal ${\beta }_{\sigma }$ for FC and ${\beta }_{\sigma }$ for BP was not statistically significant for any of the experiments (GNG: *r* =  − 0.03, *p* = 0.70; TAB: *r* = 2*e *− 3, *p* = 0.98; RS: *r* = 0.10, *p* = 0.15). The correlation between average nodal ${\beta }_{\sigma }$ for FC and ${\beta }_{\sigma }$ for GMD was positive and significant for the three experiments (GNG: *r* = 0.32, *p* = 1*e *− 7; TAB: *r* = 0.32, *p* = 2*e *− 7; RS: *r* = 0.1, *p* = 0.02). These relationships are shown in Fig. 4.

### Effect of using GSR

**Fig. 5:**
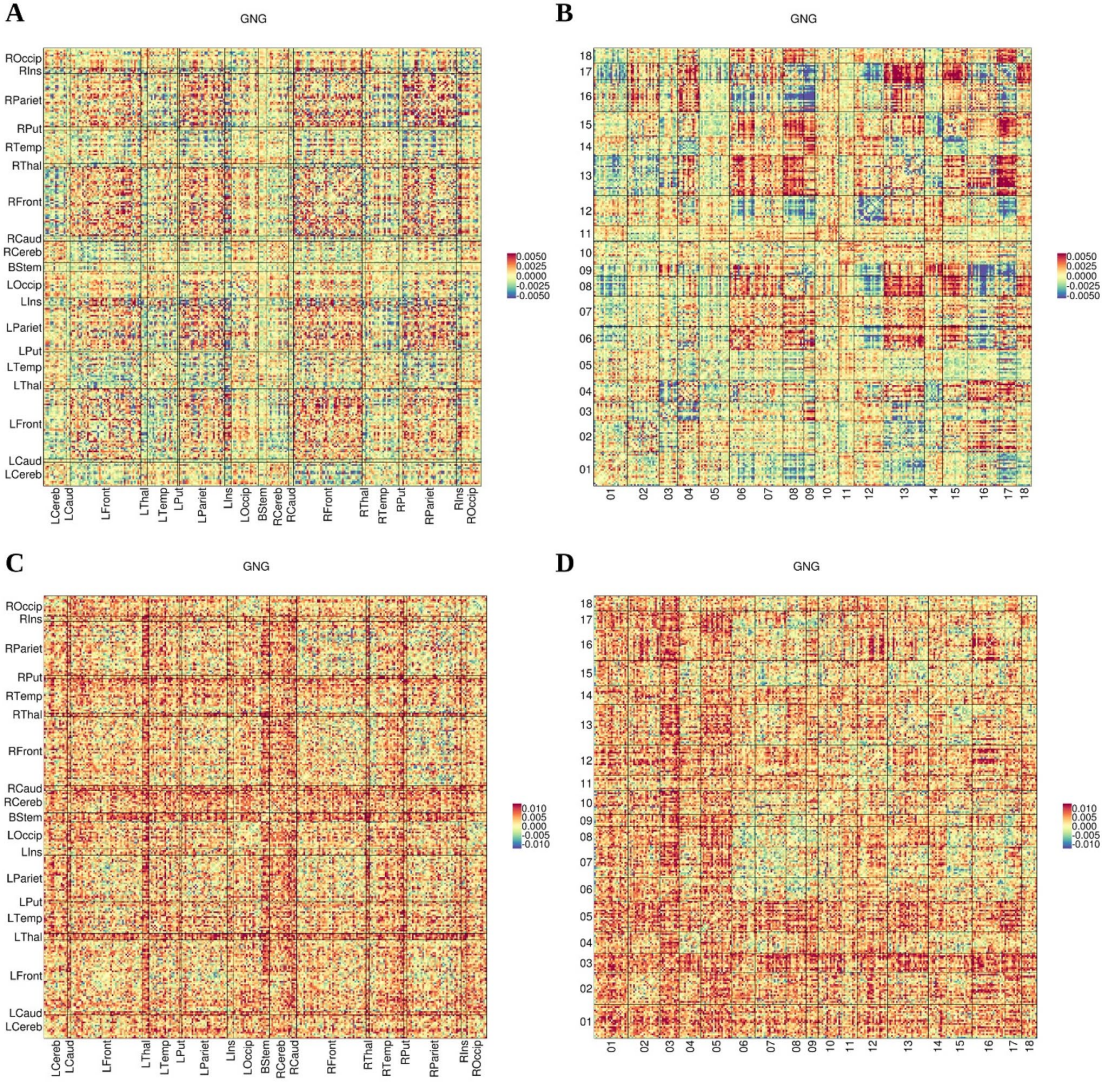
Estimates of aging effects across the connectome for data processed using GSR. For each connection between a pair of nodes in the parcellation, we show the estimates of ${\beta }_{\mu }$ (aging effects on mean FC; **a**, **b**) and ${\beta }_{\sigma }$ (aging effects on standard deviation of FC; **c**, **d**) for the GNG task with GSR. In **a** and **c**, connections are laid out in matrices that group the corresponding nodes in 19 anatomical regions: brain stem (BStem), bilateral thalamus (LThal/RThal), putamen (LPut/RPut), caudate (LCaud/RCaud), insula (LIns/RIns), cerebellum (LCereb/RCereb), and the temporal (LTemp/RTemp), parietal (LPariet/RPariet), frontal (LFront/RFront) and occipital (LOccip/ROccip) lobes. In **b** and **d**, the matrices are arranged by the networks in the 20-network BrainMap decomposition (see Supplementary Table 1 for numbering and a short description of the networks). The estimates for the other two experiments are displayed in Supplementary Figure S5 (TAB) and Supplementary Figure S6 (RS)

Applying GSR shifted ${\beta }_{\mu }$ toward positive values, centering its distribution around zero. The proportion of positive ${\beta }_{\sigma }$ values remained comparable with that for the data without GSR. The estimates of aging effects were analogously consistent across tasks with respect to the data processed without GSR (Table 2) and the distribution of aging effects still showed patterns that were constrained by anatomy and network allegiance (cf. Figs. 3 and 5 for GNG experiment). Regarding aging effects on FC and aging effects on BP or GMD, the most remarkable repercussion of using GSR was a large increase in the magnitude of the negative association between average nodal ${\beta }_{\mu }$ for FC and ${\beta }_{\mu }$ for GMD, which became clearly significant in the three datasets (Table 3). More details about the consequences of using GSR are presented in the Supplementary Material.

## Discussion

Aging leads to increases in the dissimilarity between the connectomes of younger and older subjects, and among those of older individuals. However, how the contributions to this dissimilarity are distributed anatomically and which their candidate neurobiological mechanisms are has not been characterized previously. In a sample of 30 younger and 30 older healthy adults, we examined aging effects on FC estimates across the connectome, separating components representing mean and standard deviation of FC across subjects. We showed that this decomposition is to a certain degree stable across tasks and it allowed us to map the source of similarity of functional connectomes, within and between age groups, to connections between anatomical structures and previously identified networks. Finally, we show that aging effects on FC were significantly related to aging effects on D1 BP and on gray matter structure across brain regions, and these relationships were independent from each other. The main associations observed were present in data from two fMRI tasks and a resting-state scan.

The starting point of the present investigation are the observations that: (1) the functional connectomes of individuals of different ages tend to be less similar than those of individuals of comparable age, and (2) functional connectomes decrease in similarity with increasing age (i.e., connectomes of older adults are less similar than those of younger adults). To disentangle the origins of these two phenomena, we modelled not only aging effects on mean FC, but also on interindividual variance of FC, which is a novel aspect of the current study. Analyzing the consequence of removing connections ordered by |${\beta }_{\mu }$| or |${\beta }_{\sigma }$| (see Supplementary Material) showed that, as expected, ${\beta }_{\mu }$ had a larger role in explaining the loss of similarity between age groups, whereas ${\beta }_{\sigma }$ was more important in accounting for the loss of similarity among older participants. Interestingly, connections with large |${\beta }_{\mu }$| also contributed to increase connectome similarity among older adults, which indicates that differences in those connections may drive the pattern of connectivity from a younger archetypal configuration to an older one, with higher variability of older subject connectomes around their archetypal configuration. Although the effect of the two parameters was not fully independent, this decomposition allowed us to map to individual connections the origin of both the loss of similarity between individuals of different ages and the decrease in similarity with increasing age.

We observed large heterogeneity in aging effects across functional connections, with both increases and decreases in mean FC with age (Allen et al. 2011; Geerligs et al. 2015b). Negative age-related differences were, however, more frequent than positive ones (when not applying GSR), with the largest effects seen in fronto-temporal, fronto-cerebellar and temporo-parietal connections. FC variance displayed a general tendency to increase with age; this global age-related increase in standard deviation of FC could be, partially or totally, the consequence of altered vascular function or neurovascular coupling in older participants rather than neural activity per se (D’Esposito et al. 2003; Tsvetanov et al. 2015). The anatomical distribution of aging effects on standard deviation was quite heterogeneous, with larger effects in connections involving occipital regions, thalamus, and cerebellum. Arranging the connections by intrinsic connectivity networks showed more marked patterns of increases and decreases than arranging them by anatomical structure, reflecting the known high interdependence of the neural ensembles within these networks. Prominent aging-related reductions in mean FC, indexed by ${\beta }_{\mu }$, were found for instance in connections involving temporal, default mode and limbic nodes. By contrast, the largest increases in FC variance, indexed by ${\beta }_{\sigma }$, affected preferentially sensory and motor networks. We need to be careful to avoid overinterpreting the anatomical and network distribution of aging effects because of the random variation in ${\beta }_{\mu }$ or ${\beta }_{\sigma }$ produced by sampling variation and measurement noise. However, the fact that the mentioned patterns were repeatable across experiments (see Fig. 3 and Supplementary Figures S3-4) and the relationships we present with independent measurements of GMD, and BP, indicate that they were not merely a product of the latter, but a more detailed, reliable analysis of the effects in specific networks would possibly require a considerably larger dataset.

Intriguingly, losses in FC across regions were reliably related to losses in DA D1 BP, as measured with PET. DA and FC have been associated in particular connections (Honey et al. 2003; Rieckmann et al. 2010; Wallace et al. 2011; Nyberg et al. 2016); our investigation shows that the magnitude of age differences in DA availability is related across regions with differences in (average nodal) FC, pointing at dopaminergic decline as a potential contributor to the observed functional connectome differences between younger and older adults.

We also found evidence of a negative association between aging effects on FC and GMD, such that FC decreased less for those nodes showing stronger effects of aging on GMD, and which was greatly magnified when processing the data with GSR. The reason for this negative relationship remains unknown, although it may be interpreted in compensatory terms, where reduced gray matter integrity leads to the recruitment of new regions so that behavioral functioning can be preserved (Cabeza et al. 2002). The association we detected was weak, in agreement with previous reports showing that age-related differences in FC are not fully explained by structural differences in gray matter (Damoiseaux et al. 2008; Onoda et al. 2012; Geerligs et al. 2014, 2015a). Importantly, the effects of aging on D1 BP were associated with aging effects on FC independently of aging effects on GMD, and therefore the observed relationship with BP cannot be attributed to gray matter loss, but rather suggests that this relationship has a neuromodulatory origin.

The relationship between age-related effects on standard deviation of FC was statistically significant only for GMD, in the three experiments. Correlations were positive in all cases and was conserved after GSR, implying the plausible effect that the age-related spread in the physiological process (GMD changes) which may underlie the observed age differences, is accompanied by an increase in FC variance.

Previous research has shown that functional connectivity reflects both trait and state aspects of the individual, with considerable variation across tasks but also stable features (Cole et al. 2014; Geerligs et al. 2015b), and inter-individual differences in brain responses during task being to a large extent predictable from measurements at rest (Tavor et al. 2016). Most relationships presented here were consistent across the three experiments, and may thus reflect between-group differences, produced by aging processes, in trait-like, functional-architecture components.

Reliance on cross-sectional data is a limitation of our study and most other work addressing the relationship between aging and FC. Even though we use terms like age-related decline for the sake of explanatory simplicity, it is important to bear in mind that cohort effects may confound cross-sectional, as opposed to longitudinal, data, as well as affording reduced sensitivity (Nyberg et al. 2010). In particular, low-performing subjects may be less likely to be selected for study due to poor health or mortality. Although this may be a source of bias, note that it should produce smaller rather than larger heterogeneity in aging. It is also important to stress that the effects we report should not be interpreted as a mediation. Mediation analyses should ideally be performed on longitudinal data (unfortunately not available in the present study), as those performed on cross-sectional data are known to be prone to bias in the estimation of the parameters (Lindenberger et al. 2011; Laughlin et al. 2018). A further limitation of the present study is that we did not collect measures of education, health or fluid intelligence, and therefore we cannot rule out completely that group differences in these may be confounding the differences in neural measures we found.

In-scanner motion, which may artifactually increase the degree of correlation between BOLD time-courses, deserves some cautionary comments. For the GNG, not even an aggressive correction (FD threshold at 0.3 mm) removed the difference in mean FD between younger and older participants. Although a FD threshold above 0.2 mm will leave demonstrable motion artifacts (Power et al. 2014), such a restrictive threshold would have excluded too much data in our study. Other investigations on the effects of aging on FC have used less or equally strict thresholds than the one we used here (Chan et al. 2014; Geerligs et al. 2014, 2015a; Ferreira et al. 2016). It is worth pointing out that there exist a number of motion-correction strategies available (Ciric et al. 2017; Parkes et al. 2018), and beyond censoring methods like we have used, ICA-based methods (Pruim et al. 2015) can be very effective at removing motion-related artifacts. Besides censoring high-motion volumes, as a further control we regressed out a quadratic function of the motion parameters and their derivatives (Satterthwaite et al. 2013; Power et al. 2014). Using GSR had an impact on the estimates of aging effects and weakened but did not eliminate the relationships we report with BP. GSR may remove neural signal of interest (Murphy and Fox 2017), and it seems unlikely that these associations would arise solely as a product of motion, but with the current data we cannot completely rule out this possibility. Differences in the impact of GSR between the three experiments are likely to be, at least in part, the result of the different activation profiles associated with their particular cognitive requirements. GSR induces a negative shift in FC (Murphy et al. 2009; Li et al. 2019), and the resulting estimates can be interpreted as relative to the whole-brain signal (Yeo et al. 2015), which may account for the shift observed in aging effects when applying GSR. While the derived aging effects are less affected by motion and physiological artifacts, their interpretation becomes less straightforward.

We focus on the GNG data because it had a long acquisition time and the largest number of subjects after exclusion of those with excessive motion, but we show that our main conclusions are preserved in the TAB and RS experiments, for which there was no statistically significant age-group difference in a summary measure of in-scanner motion. We note that the scanning parameters were the same in the three experiments, with the only exception being the number of volumes. Owing to their greater scanning length compared to the RS, the task datasets allowed us to obtain more robust estimates of aging effects.

## Conclusion

We have mapped differences between younger and older subjects in mean and standard deviation for a comprehensive set of functional connections, and shown that low similarity between the connectomes of younger and older individuals reflects age-related changes in mean FC, whereas the loss of similarity between the connectomes of older participants is associated with age-related differences in FC variance. We observed that age-related alterations in D1 receptor availability were related to differences in mean FC controlling for gray matter morphology differences. Additionally, age-related differences in variance of FC were related to differences in variance of GMD. These findings indicate that DA decline may contribute to the loss of similarity between the connectomes of younger and older subjects, whereas differential patterns of aging-related GMD loss mainly promote the increased dissimilarity among the connectomes of older adults.

## Supplementary Information

Below is the link to the electronic supplementary material.

Supplementary file1 (DOCX 7038 KB)

## Data Availability

Processed data associated with this manuscript are available at https://data.mendeley.com/datasets/5s8bjrz7jt/draft?a=41aba1c3-e7dc-44f0-a5a8-c5f193f21ab7.

## Code availability

Code used for data processing and analysis is available at https://github.com/benjamingarzon/DAandConnectomes.

## References

[CR1] Allen EA, Erhardt EB, Damaraju E (2011). A baseline for the multivariate comparison of resting-state networks. Front Syst Neurosci.

[CR2] Andrews-Hanna JR, Snyder AZ, Vincent JL (2007). Disruption of large-scale brain systems in advanced aging. Neuron.

[CR3] Ashburner J (2007). A fast diffeomorphic image registration algorithm. Neuroimage.

[CR4] Ashburner J, Friston KJ (2000). Voxel-based morphometry—the methods. Neuroimage.

[CR5] Ashburner J, Friston KJ (2005). Unified segmentation. Neuroimage.

[CR6] Bäckman L, Lindenberger U, Li SC, Nyberg L (2010). Linking cognitive aging to alterations in dopamine neurotransmitter functioning: recent data and future avenues. Neurosci Biobehav Rev.

[CR7] Biswal B, Yetkin FZ, Haughton VM, Hyde JS (1995). Functional connectivity in the motor cortex of resting human brain using echo-planar MRI. Magn Reson Med.

[CR8] Cabeza R, Anderson ND, Locantore JK, McIntosh AR (2002). Aging gracefully: compensatory brain activity in high-performing older adults. Neuroimage.

[CR9] Carbonell F, Nagano-saito A, Leyton M (2014). Dopamine precursor depletion impairs structure and efficiency of resting state brain functional networks. Neuropharmacology.

[CR10] Chan MY, Park DC, Savalia NK (2014). Decreased segregation of brain systems across the healthy adult lifespan. Proc Natl Acad Sci.

[CR11] Chowdhury R, Guitart-masip M, Lambert C (2013). Dopamine restores reward prediction errors in old age. Nat Neurosci.

[CR12] Ciric R, Wolf DH, Power JD (2017). Benchmarking of participant-level confound regression strategies for the control of motion artifact in studies of functional connectivity. Neuroimage.

[CR13] Cole DM, Beckmann CF, Oei NYL (2013). Differential and distributed effects of dopamine neuromodulations on resting-state network connectivity. Neuroimage.

[CR14] Cole MW, Bassett DS, Power JD (2014). Intrinsic and task-evoked network architectures of the human brain. Neuron.

[CR15] D’Esposito M, Deouell LY, Gazzaley A (2003). Alterations in the BOLD fMRI signal with ageing and disease: a challenge for neuroimaging. Nat Rev Neurosci.

[CR16] Damoiseaux JS, Beckmann CF, Arigita EJS (2008). Reduced resting-state brain activity in the ‘“default network”’ in normal aging. Cereb Cortex.

[CR17] de Boer L, Axelsson J, Riklund K (2017). Attenuation of dopamine-modulated prefrontal value signals underlies probabilistic reward learning deficits in old age. Elife.

[CR18] de Boer L, Axelsson J, Chowdhury R (2019). Dorsal striatal dopamine D1 receptor availability predicts an instrumental bias in action learning. Proc Natl Acad Sci USA.

[CR19] Durstewitz D, Seamans JK (2008). The dual-state theory of prefrontal cortex dopamine function with relevance to catechol-*O*-methyltransferase genotypes and schizophrenia. Biol Psychiatry.

[CR20] Fandakova Y, Lindenberger U, Shing YL (2015). Neurobiology of Aging Maintenance of youth-like processing protects against false memory in later adulthood. Neurobiol Aging.

[CR21] Ferreira LK, Regina ACB, Kovacevic N (2016). Aging effects on whole-brain functional connectivity in adults free of cognitive and psychiatric disorders. Cereb Cortex.

[CR22] Finn ES, Shen X, Scheinost D (2015). Functional connectome fingerprinting: identifying individuals using patterns of brain connectivity. Nat Neurosci.

[CR23] Finn ES, Scheinost D, Finn DM (2017). Can brain state be manipulated to emphasize individual differences in functional connectivity?. Neuroimage.

[CR24] Fischl B, Salat DH, Busa E (2002). Whole brain segmentation: automated labeling of neuroanatomical structures in the human brain. Neuron.

[CR25] Fisher R (1921). On the “probable error” of a coefficient of correlation deduced from a small sample. Metron.

[CR26] Fox PT, Laird AR, Fox SP (2005). BrainMap taxonomy of experimental design: description and evaluation. Hum Brain Mapp.

[CR27] Geerligs L, Maurits NM, Renken RJ, Lorist MM (2014). Reduced specificity of functional connectivity in the aging brain during task performance. Hum Brain Mapp.

[CR28] Geerligs L, Renken R, Saliasi E (2015). A brain-wide study of age-related changes in functional connectivity. Cereb Cortex.

[CR29] Geerligs L, Rubinov M, Cam-CAN HRN (2015). State and trait components of functional connectivity: individual differences vary with mental state. J Neurosci.

[CR30] Groves AR, Smith SM, Fjell AM (2012). Benefits of multi-modal fusion analysis on a large-scale dataset: life-span patterns of inter-subject variability in cortical morphometry and white matter microstructure. Neuroimage.

[CR31] Guitart-Masip M, Fuentemilla L, Bach DR (2011). Action dominates valence in anticipatory representations in the human striatum and dopaminergic midbrain. J Neurosci.

[CR32] Guitart-Masip M, Huys QJM, Fuentemilla L (2012). Go and no-go learning in reward and punishment: interactions between affect and effect. Neuroimage.

[CR33] Guitart-Masip M, Economides M, Huys QJM (2014). Differential, but not opponent, effects of l-DOPA and citalopram on action learning with reward and punishment. Psychopharmacology.

[CR34] Hall H, Sedvall G, Magnusson O (1994). Distribution of D1- and D2-dopamine receptors, and dopamine and its metabolites in the human brain. Neuropsychopharmacology.

[CR35] Honey GD, Suckling J, Zelaya F (2003). Dopaminergic drug effects on physiological connectivity in a human cortico-striato-thalamic system. Brain.

[CR36] Hout MC, Godwin HJ, Fitzsimmons G (2016). Using multidimensional scaling to quantify similarity in visual search and beyond. Atten Percept Psychophys.

[CR37] Jenkinson M, Bannister P, Brady M, Smith S (2002). Improved optimization for the robust and accurate linear registration and motion correction of brain images. Neuroimage.

[CR38] Kennedy KM, Erickson KI, Rodrigue KM (2009). Age-related differences in regional brain volumes: a comparison of optimized voxel-based morphometry to manual volumetry. Neurobiol Aging.

[CR39] Laird AR, Fox PM, Eickhoff SB (2011). Behavioral interpretations of intrinsic connectivity networks. J Cogn Neurosci.

[CR40] Laughlin KDO, Martin MJ, Ferrer E (2018). Cross-sectional analysis of longitudinal mediation processes cross-sectional analysis of longitudinal mediation processes. Multivariate Behav Res.

[CR41] Lebedev AV, Nilsson J, Lövdén M (2018). Working memory and reasoning benefit from different modes of large-scale brain dynamics in healthy older adults. Hum Brain Mapp.

[CR42] Li S-C, Lindenberger U (1999). Cross-level unification: a computation exploration of the link between deterioration of neurotransmitter systems and dedifferentiation of cognitive abilities in old age. Cognitive neuroscience of memory.

[CR43] Li S-C, Sikström S (2002). Integrative neurocomputational perspectives on cognitive aging, neuromodulation, and representation. Neurosci Biobehav Rev.

[CR44] Li S-C, Lindenberger U, Sikström S (2001). Aging cognition: from neuromodulation to representation. Trends Cogn Sci.

[CR45] Li J, Kong R, Orban C (2019). Global signal regression strengthens association between resting-state functional connectivity and behavior. Neuroimage.

[CR46] Lindenberger U, von Oertzen T, Ghisletta P, Hertzog C (2011). Cross-sectional age variance extraction: What’s change got to do with it?. Psychol Aging.

[CR47] Logan J, Fowler JS, Volkow ND (1990). Graphical analysis of reversible radioligand binding from time-activity measurements applied to [*N*-11*C*-methyl]-(–)-Cocaine PET studies in human subjects. J Cereb Blood Flow Metab.

[CR48] Mevel K, Landeau B, Fouquet M (2013). Age effect on the default mode network, inner thoughts, and cognitive abilities. Neurobiol Aging.

[CR49] Mowinckel AM, Espeseth T, Westlye LT (2012). Network-specific effects of age and in-scanner subject motion: a resting-state fMRI study of 238 healthy adults. Neuroimage.

[CR50] Murphy K, Fox MD (2017). Towards a consensus regarding global signal regression for resting state functional connectivity MRI. Neuroimage.

[CR51] Murphy K, Birn RM, Handwerker DA (2009). The impact of global signal regression on resting state correlations: Are anti-correlated networks introduced?. Neuroimage.

[CR52] Nyberg L, Salami A, Andersson M (2010). Longitudinal evidence for diminished frontal cortex function in aging. PNAS.

[CR53] Nyberg L, Lövdén M, Riklund K (2012). Memory aging and brain maintenance. Trends Cogn Sci.

[CR54] Nyberg L, Karalija N, Salami A (2016). Dopamine D2 receptor availability is linked to hippocampal–caudate functional connectivity and episodic memory. PNAS.

[CR55] Onoda K, Ishihara M, Yamaguchi S (2012). Decreased functional connectivity by aging is associated with cognitive decline. J Cogn Neurosci.

[CR56] Parkes L, Fulcher B, Yücel M, Fornito A (2018). An evaluation of the efficacy, reliability, and sensitivity of motion correction strategies for resting-state functional MRI. Neuroimage.

[CR57] Power JD, Barnes K, Snyder A (2012). Spurious but systematic correlations in resting state functional connectivity MRI arise from head motion. Neuroimage.

[CR58] Power JD, Mitra A, Laumann TO (2014). Methods to detect, characterize, and remove motion artifact in resting state fMRI. Neuroimage.

[CR59] Pruim RHR, Mennes M, Van RD (2015). ICA-AROMA: a robust ICA-based strategy for removing motion artifacts from fMRI data. Neuroimage.

[CR60] Raz N, Lindenberger U, Rodrigue KM (2005). Regional brain changes in aging healthy adults: general trends, individual differences and modifiers. Cereb Cortex.

[CR61] Reuter M, Tisdall MD, Qureshi A (2015). Head motion during MRI acquisition reduces gray matter volume and thickness estimates. Neuroimage.

[CR62] Rieckmann A, Fischer H, Bäckman L (2010). Activation in striatum and medial temporal lobe during sequence learning in younger and older adults: relations to performance. Neuroimage.

[CR63] Rieckmann A, Karlsson S, Fischer H, Bäckman L (2011). Caudate dopamine D1 receptor density is associated with individual differences in frontoparietal connectivity during working memory. J Neurosci.

[CR500] Ross S, Stearns C (2010) SharpIR: white paper [Internet]. http://www3.gehealthcare.co.uk/~/media/downloads/uk/education/pet%20white%20papers/mi_emea_sharpir_white_paper_pdf_092010_doc0852276.pdf?Parent=%7BB66C9E27-1C45-4F6B-BE27-D2351D449B19%7D. Accessed 9 Jan 2017

[CR64] Rosenberg MD, Finn ES, Scheinost D (2015). A neuromarker of sustained attention from whole-brain functional connectivity. Nat Neurosci.

[CR65] Salami A, Pudas S, Nyberg L (2014). Elevated hippocampal resting-state connectivity underlies deficient neurocognitive function in aging. Proc Natl Acad Sci.

[CR66] Salami A, Wahlin A, Kaboodvand N (2016). Longitudinal evidence for dissociation of anterior and posterior MTL resting-state connectivity in aging: links to perfusion and memory. Cereb Cortex.

[CR67] Satterthwaite TD, Elliott MA, Gerraty RT (2013). An improved framework for confound regression and filtering for control of motion artifact in the preprocessing of resting-state functional connectivity data. Neuroimage.

[CR68] Savalia NK, Agres PF, Chan MY (2017). Motion-related artifacts in structural brain images revealed with independent estimates of in-scanner head motion. Hum Brain Mapp.

[CR69] Servan-Schreiber D, Printz H, Cohen JD (1990). A network model of catecholamiine effects: gain, signal-to-noise ratio, and behavior. Science (80–).

[CR70] Shen X, Tokoglu F, Papademetris X, Constable RT (2013). Groupwise whole-brain parcellation from resting-state fMRI data for network node identification. Neuroimage.

[CR71] Smith SM, Fox PT, Miller KL (2009). Correspondence of the brain’s functional architecture during activation and rest. PNAS.

[CR72] Stumme J, Jockwitz C, Hoffstaedter F (2020). Functional network reorganization in older adults: Graph-theoretical analyses of age, cognition and sex. Neuroimage.

[CR73] Tavor I, Jones OP, Mars RB (2016). Task-free MRI predicts individual differences in brain activity during task performance. Science (80–).

[CR74] Tsvetanov KA, Henson RNA, Tyler LK (2015). The effect of ageing on fMRI: correction for the confounding effects of vascular reactivity evaluated by joint fMRI and MEG in 335 adults. Hum Brain Mapp.

[CR75] Van Dijk KRA, Sabuncu MR, Buckner RL (2012). The influence of head motion on intrinsic functional connectivity MRI. Neuroimage.

[CR76] Wallace DL, Vytlacil JJ, Nomura EM (2011). The dopamine agonist bromocriptine differentially affects fronto-striatal functional connectivity during working memory. Front Hum Neurosci.

[CR77] Yan (2010). DPARSF: a MATLAB toolbox for “pipeline” data analysis of resting-state fMRI. Front Syst Neurosci.

[CR78] Yeo BTT, Tandi J, Chee MWL (2015). Functional connectivity during rested wakefulness predicts vulnerability to sleep deprivation. Neuroimage.

