# Supplementary material for: Role of dopamine and gray matter density in aging effects and individual differences of functional connectomes
Source: Brain Struct Funct. 2021 Jan 9;226(3):743–58. doi: 10.1007/s00429-020-02205-4 (PMC7981334; doi:10.1007/s00429-020-02205-4)
Supplement: Supplementary file 1 — Supplementary file1 (DOCX 7038 KB) [file 429_2020_2205_MOESM1_ESM.docx]

# SUPPLEMENTARY MATERIAL

## Effect of connection removal

We expected that $\beta_{\mu}$ would affect more strongly the similarity between the functional connectomes of younger and older subjects, whereas the effect of $\beta_{\sigma}$ would be larger for the similarity between the functional connectomes of older subjects. To test this assumption, we first ordered connections by decreasing magnitude of the absolute value of $\beta_{\mu}$. We then removed the first *N_c_* connections, in that order and varying *N_c_*, and computed the average similarity between all possible pairs of old subjects, as well as between all pairs including one younger and one older subject. We varied *N_c_* from zero to the total number of connections, with a total of 20 equidistant values, and plotted the average connectome similarity for each value of *N_c_*. We repeated the same procedure this time ordering the connections by the absolute value of $\beta_{\sigma}$, in decreasing order. These analyses were repeated 20 times for random orderings of removed connections to show that the similarity values obtained when removing the connections in order of |$\beta_{\mu}$| and |$\beta_{\sigma}$| were very unlikely to occur by chance.

Indeed, removing connections with large |$\beta_{\mu}$| resulted in a higher average similarity between younger and older participants (Supplementary Figure S2 A, red trace) compared to removing connections at random (blue traces). In contrast, the average similarity between older subjects became lower when removing connections randomly (Supplementary Figure S2 B). Therefore, connections with large aging effects on mean FC contributed most preeminently to make the connectomes of younger and older more dissimilar, and at the same time make the connectomes of older persons more alike. In the case where the removed connections were ordered by the value of |$\beta_{\sigma}$|, far fewer connections needed to be removed to obtain an average similarity that was higher than for random removal when computing the average similarity among older subjects (Supplementary Figure S2 D) than when computing the average similarity between younger and older subjects (Supplementary Figure S2 C). Hence, connections with large ${|\beta}_{\mu}|$ clearly appeared to be driving the loss of similarity between functional connectomes of younger and older subjects while simultaneously explaining the similarity between older subjects. However, those with large ${|\beta}_{\sigma}|$ had a more conspicuous effect on the similarity of connectomes of pairs of older participants than on younger-older similarity (although this effect was less marked than that of $\beta_{\mu}$ on younger-older differences). Note that this analysis was not meant to test a hypothesis, but rather to illustrate how the estimated model parameters $\beta_{\mu}$ and $\beta_{\sigma}$ allow mapping the individual contribution of the different anatomical connections to connectome similarity patterns between age groups, and to demonstrate the equivalence of these representations.

## Effect of using GSR

Including GSR in the fMRI preprocessing pipeline resulted in a shift of $\beta_{\mu}$ toward positive values (GNG: 48.9 % of negative connections; TAB: 49.0 %; RS: 48.1 %), such that its distribution became approximately centered around zero. The proportion of positive $\beta_{\sigma}$ values remained comparable with that for the data without GSR (GNG: 73.3 % of connections; TAB: 78.8 %; RS: 69.8 %). The estimates of aging effects were analogously consistent across tasks with respect to the data processed without GSR (Table 2).

Similarities between younger and older subjects were still lower than for pairs of younger or older subjects. The difference between the average younger-younger similarity and the average older-older similarity was 0.09 (two sample t-test, t = 19.8, p < 2e-10). The difference between average older-older similarity and older-younger similarity was 0.05 (two sample t-test, t = 11, p < 2e-10). The distribution of aging effects still showed patterns that were constrained by anatomy and network allegiance (cf. Figures 3 and 4 for GNG, Supplementary Figures S3 and S4 for TAB and Supplementary Figures S5 and S6 for RS).

When comparing aging effects on FC and aging effects on BP or GMD, the most remarkable repercussion of using GSR was a large increase in the magnitude of the negative association between average nodal $\beta_{\mu}$ for FC and $\beta_{\mu}$ for GMD (GNG: *r* = -0.51, p < 1e-10; TAB: *r* = -0.48, p < 1e-10; RS: *r* = -0.52, p < 1e-10), which became clearly significant in the three datasets. Average nodal $\beta_{\mu}$ for FC was still significantly correlated with $\beta_{\mu}$ for BP (GNG: *r* = 0.21, p = 0.001; TAB: *r* = 0.18, p = 0.006; RS: *r* = 0.24, p = 2e-4) even after correcting for $\beta_{\mu}$ for GMD (significant for GNG/RS and at trend level for TAB; see Table 3 for regression coefficients). Nevertheless, the correlations were smaller than before, and not significant anymore when the ROIs with the largest age-related BP losses ($\beta_{\mu}$ < - 0.01) were removed from the analysis (GNG: *r* = 0.06, p = 0.38; TAB: *r* = 0.05, p = 0.49; RS: *r* = -0.03, p = 0.70). As before, the association between average nodal $\beta_{\sigma}$ for FC and $\beta_{\sigma}$ for BP was not statistically significant for any of the experiments (GNG: *r* = -0.10, p = 0.13; TAB: *r* = -0.07, p = 0.31; RS: *r* = 0.10, p = 0.15). The correlation between average nodal $\beta_{\sigma}$ for FC and $\beta_{\sigma}$ for GMD was still positive and significant for the three experiments (GNG: *r* = 0.32, p = 9e-8; TAB: *r* = 0.23, p = 1e-4; RS: *r* = 0.24, p = 1e-4).

## SUPPLEMENTARY TABLES

**Supplementary Table 1.** **Description of the 18 coactivation networks from the BrainMap meta-analysis.**

| **Number** | **Description (Laird 2011)** | **Description (Smith 2009)** |
| --- | --- | --- |
| 01 | Limbic and medial-temporal regions |  |
| 02 | Subgenual anterior cingulate and orbitofrontal cortex | Executive control |
| 03 | Bilateral basal ganglia and thalamus |  |
| 04 | Bilateral anterior insula/frontal opercula and the anterior aspect of the body of the cingulate gyrus |  |
| 05 | Cerebellum – Brainstem |  |
| 06 | Superior and middle frontal gyri |  |
| 07 | Middle frontal gyri and superior parietal lobules |  |
| 08 | Ventral precentral gyri, central sulci, postcentral gyri, superior and inferior cerebellum | Sensorimotor |
| 09 | Superior parietal lobule |  |
| 10 | Middle and inferior temporal gyri | Lateral visual |
| 11 | Lateral posterior occipital cortices | Occipital pole visual |
| 12 | Medial posterior occipital cortices | Medial visual |
| 13 | Medial prefrontal and posterior cingulate/precuneus regions (default mode network) | Default mode network |
| 14 | Cerebellum | Cerebellum |
| 15 | Right-lateralized fronto-parietal regions | Right fronto-parietal |
| 16 | Transverse temporal gyri | Auditory |
| 17 | Dorsal precentral gyri, central sulci, postcentral gyri, superior and inferior cerebellum |  |
| 18 | Left-lateralized fronto-parietal regions | Left fronto-parietal |

Number coding and descriptions extracted from (Laird et al., 2011) and (Smith et al., 2009).

## SUPPLEMENTARY FIGURES


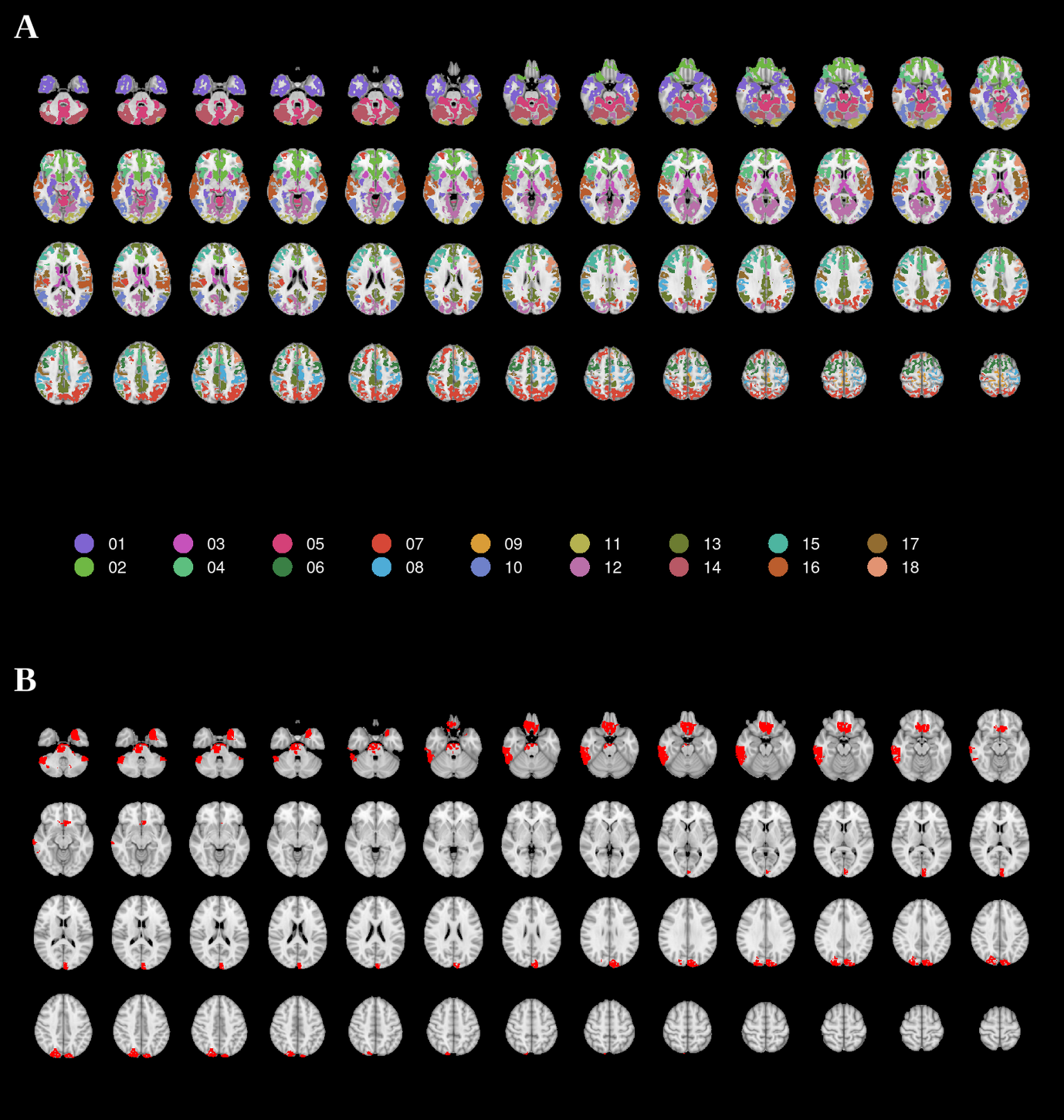


**Supplementary Figure S1. Parcellation used in the study.** A) Nodes from the parcellation (Shen et al., 2013) that were used for the analyses, labelled according to the BrainMap decomposition. The color coding refers to the networks described in Supplementary Table 1. B) For analyses, nodes in red were excluded from the original parcellation because of low signal in some of the subjects, as explained in the Materials and Methods section (those nodes do not appear in A).


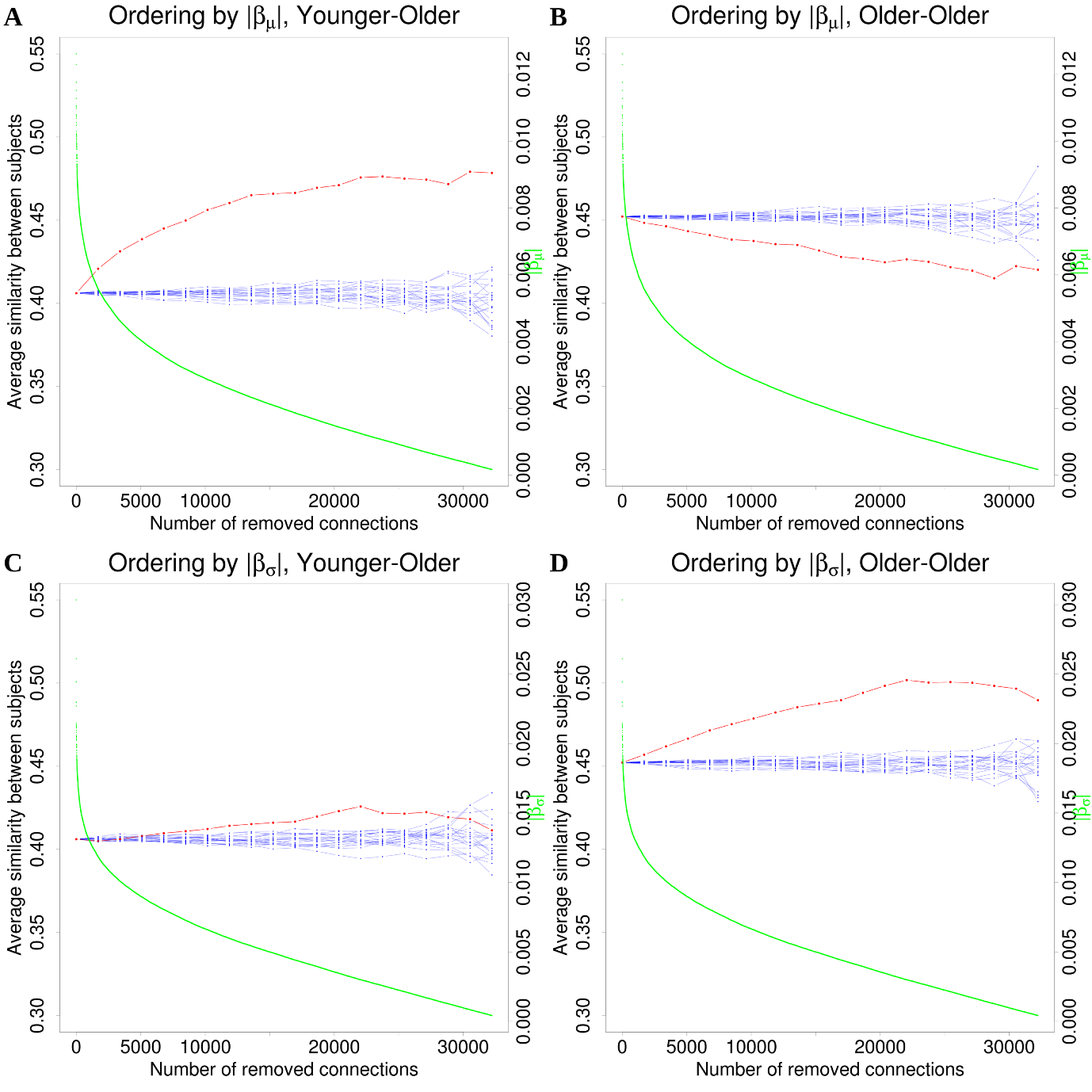


**Supplementary Figure S2. Relationships between individual aging effects and connectome similarity.** Effect of removing connections on overall similarity for younger vs younger and younger vs older subjects, ordered by decreasing |$\beta_{\mu}$| (magnitude of aging effects on mean FC; A, B), and ordered by decreasing |$\beta_{\sigma}$| (magnitude of aging effects on standard deviation of FC; C, D). The red trace shows average similarity between connectomes when removing connections ordered by the magnitude of |$\beta_{\mu}$| (A, B) or |$\beta_{\sigma}$| (C, D), and that magnitude is represented by the green trace. The blue traces show average similarity between connectomes when removing connections in a random order (repeated 20 times with different orderings, each trace corresponds to one ordering). When connections with large magnitude of their aging effects$\beta_{\mu}$,$\beta_{\sigma}$ were removed, the value of average similarity between age groups obtained was unlikely to occur by chance (most conspicuously in A and D).


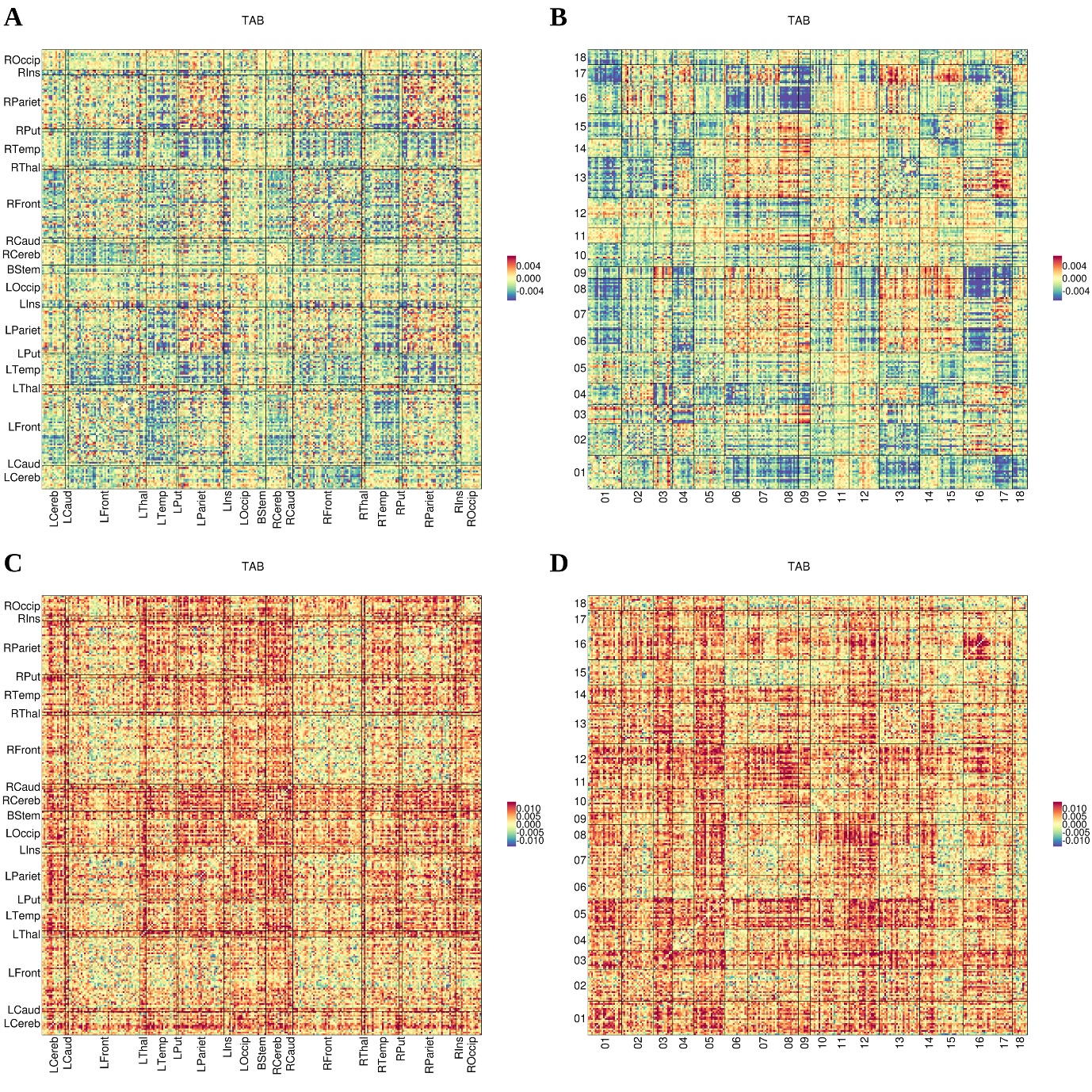


**Supplementary Figure S3. Estimates of aging effects across the connectome for the TAB experiment and data processed without using GSR.** Equivalent to Figure 3 but for the TAB experiment data.


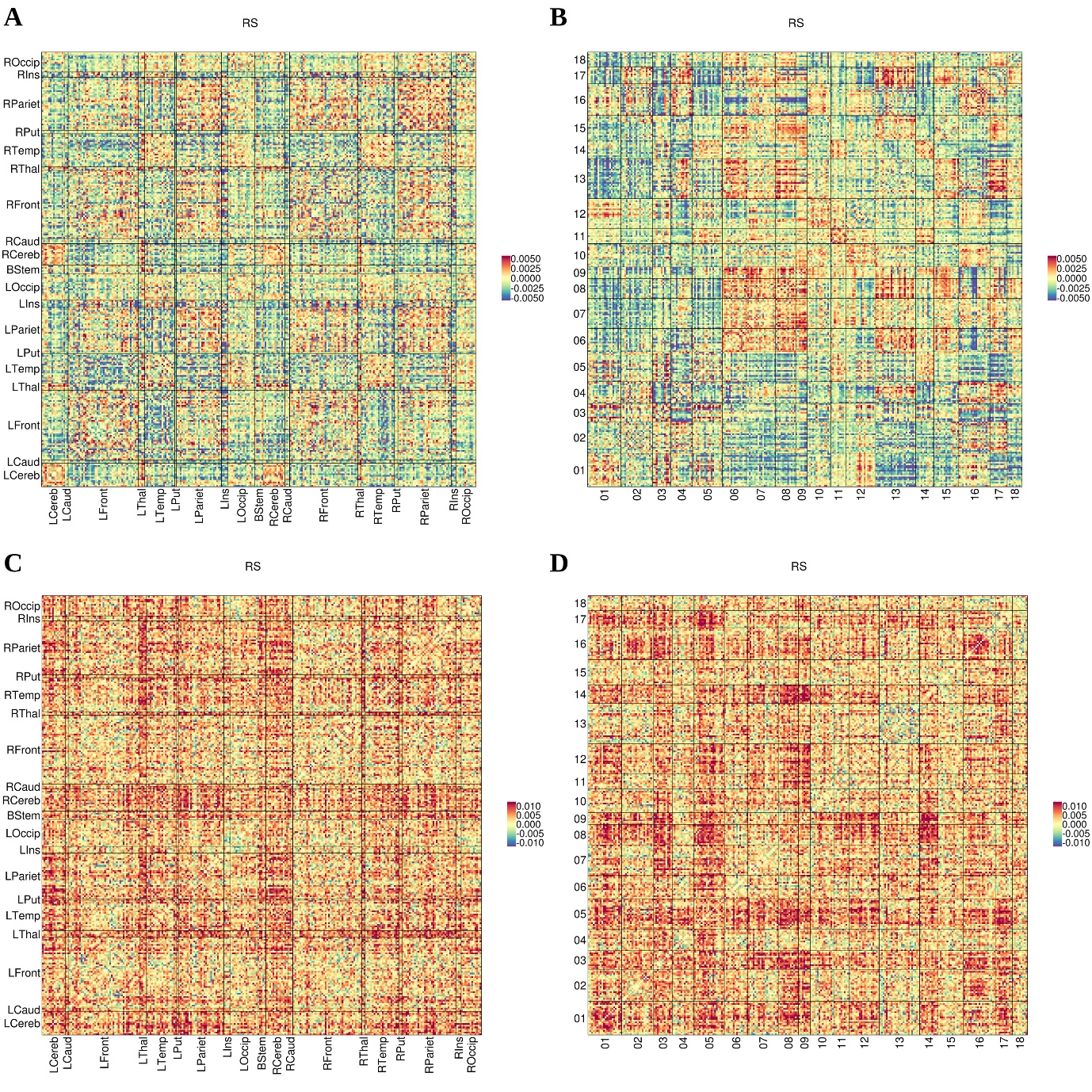


**Supplementary Figure S4. Estimates of aging effects across the connectome for the RS experiment and data processed without using GSR.** Equivalent to Figure 3 but for the RS experiment data.


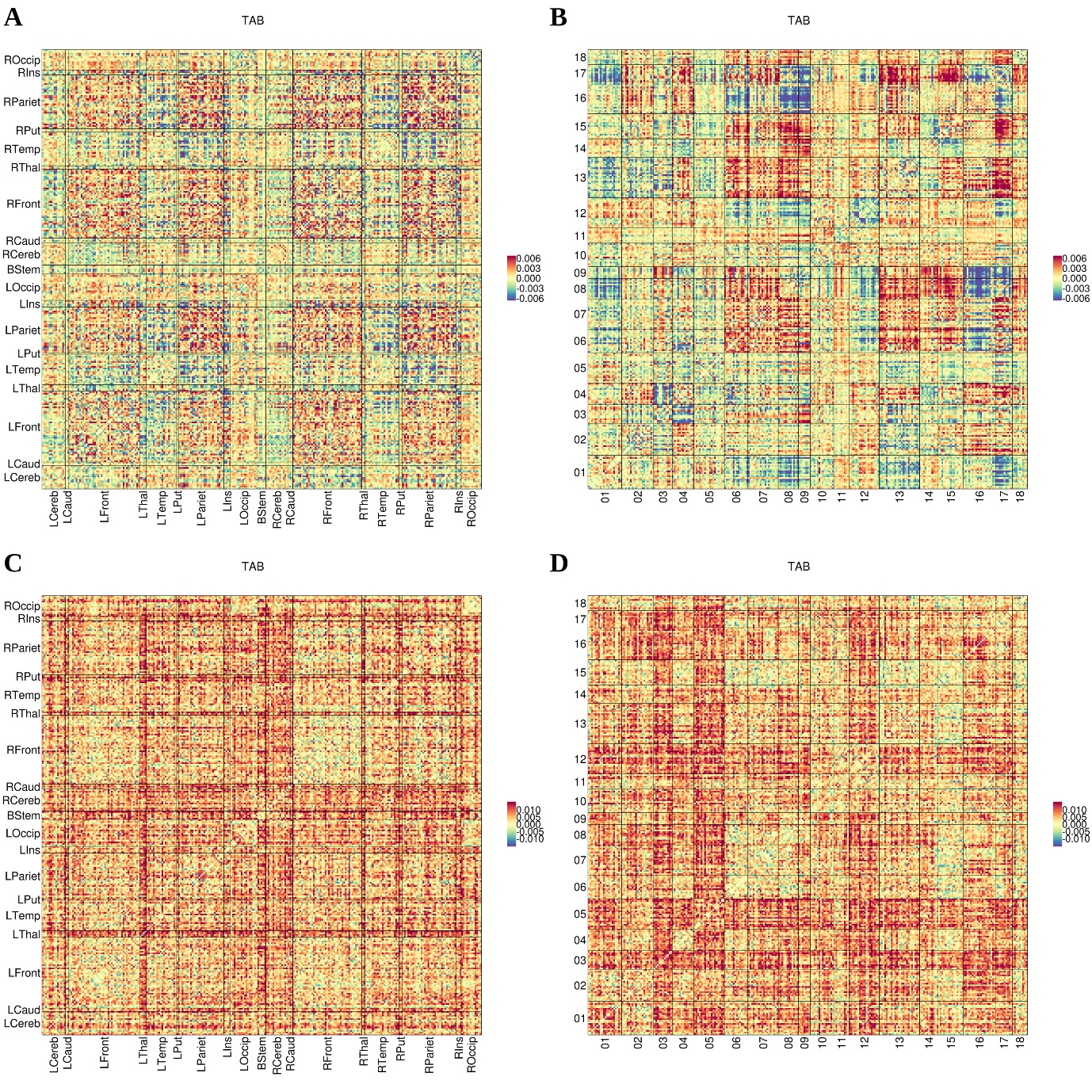


**Supplementary Figure S5. Estimates of aging effects across the connectome for the TAB experiment and data processed using GSR.** Equivalent to Figure 4 but for the TAB experiment data.


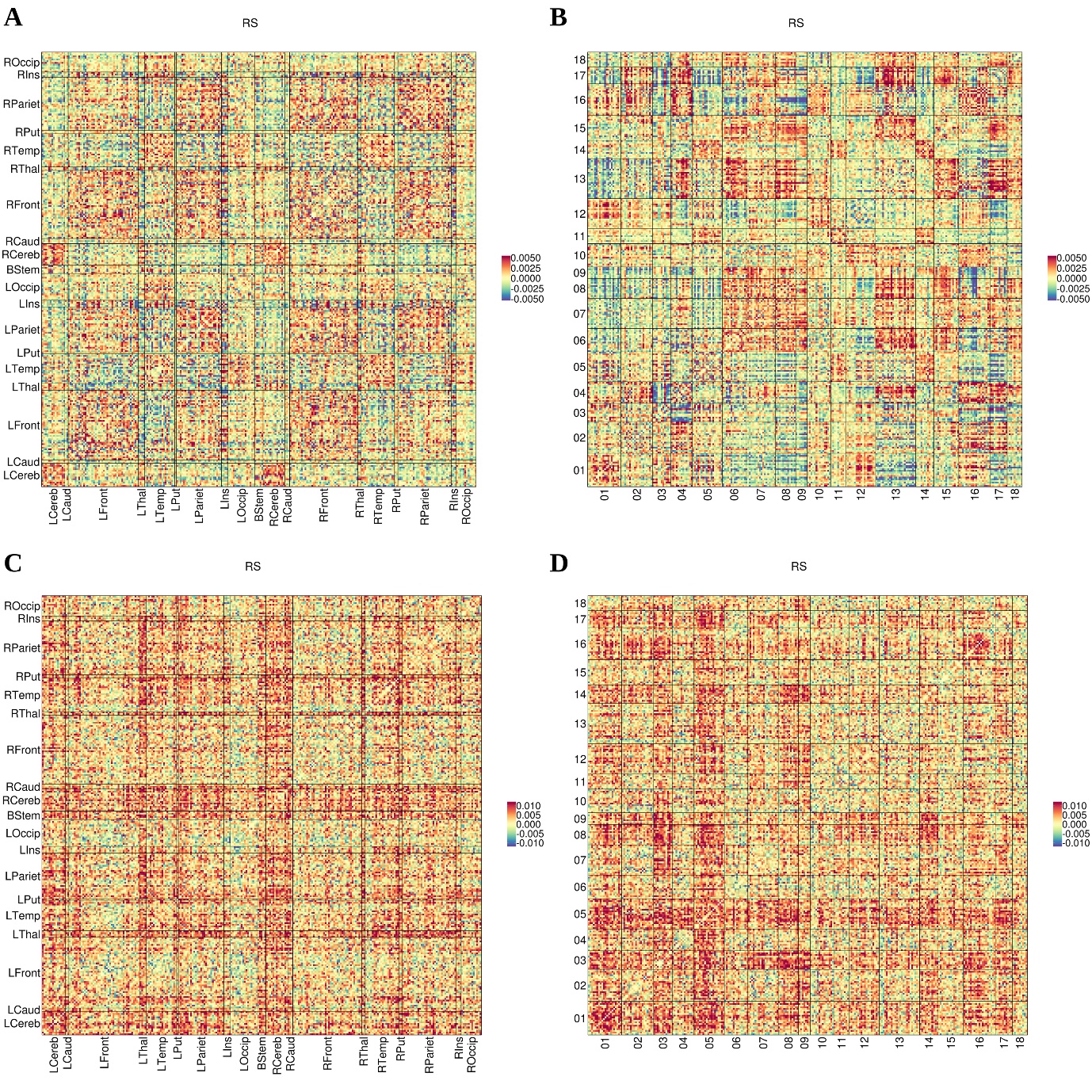


**Supplementary Figure S6. Estimates of aging effects across the connectome for the RS experiment and data processed using GSR.** Equivalent to Figure 4 but for the RS experiment data.
